# The Relative Strength of #SAT Proof Systems

**DOI:** 10.1007/s10817-026-09757-w

**Published:** 2026-07-20

**Authors:** Olaf Beyersdorff, Johannes K. Fichte, Markus Hecher, Tim Hoffmann, Lea Kasche

**Affiliations:** 1 Friedrich Schiller University, Jena, Germany; 2 University of Potsdam, Potsdam, Germany; 3 University Artois, CNRS, UMR 8188, CRIL, Lens, France; 4 Linköping University, Linköping, Sweden

**Keywords:** Model Counting, #SAT, Proof Complexity, Proof Systems, Lower Bounds, Knowledge Compilation

## Abstract

The propositional model counting problem #SAT asks to compute the number of satisfying assignments for a given propositional formula. Recently, three #SAT proof systems $\textsf{kcps}$ (knowledge compilation proof system), $\textsf{MICE}$ (model counting induction by claim extension), and $\textsf{CPOG}$ (certified partitioned-operation graphs) have been introduced with the aim to model #SAT solving and enable proof logging for solvers. A fourth system, $\textsf{CLIP}$ (circuit linear introduction proposition), is a very powerful proof system of theoretical interest. Prior to this paper, it was only known that $\textsf{CLIP}$ simulates the three other systems. All the remaining relations between the systems have been unclear and very few proof complexity results are known. We completely determine the simulation order of the four systems, establishing that $\textsf{CPOG}$ simulates both $\textsf{MICE}$ and $\textsf{kcps}$, while $\textsf{MICE}$ and $\textsf{kcps}$ are exponentially incomparable. This implies that $\textsf{CPOG}$ is strictly stronger than the other two systems.

## Introduction

The *propositional model counting problem* #SAT asks to compute the number of satisfying complete assignments for a given propositional formula [15]. The #SAT framework allows to efficiently encode and solve many real-world problems from areas such as probabilistic reasoning [3, 56], risk analysis [32, 72] and explainable artificial intelligence [4, 63]. Interestingly, #SAT is among the hardest combinatorial problems and known to be #P-complete [3, 61, 67]. To put this in relation, by Toda’s Theorem [65] any problem from the polynomial hierarchy (PH) can be solved in polynomial time by access to a #SAT oracle. In comparison, the SAT problem is on the first level of PH [25].

Over the last two decades, researchers and solver engineers improved effective *practical #SAT solving* [40] with numerous available #SAT solvers using conceptually quite different approaches. An annual competition captures current trends of solvers and novel practical algorithms, but also reveals that correctness needs to be improved [33, 34].

In contrast to these practical advances, little is known theoretically on the power and limitations of #SAT solving. In both SAT and quantified Boolean formulas (QBF), the main theoretical approach towards gauging the strength of SAT and QBF solvers is through *proof systems and proof complexity* [11, 19]. The relation between proofs and solving is important in at least two aspects.

Firstly, proof systems can *model aspects of solving*. A seminal result in this direction is that CDCL solvers – the predominant approach in SAT solving – tightly correspond to propositional resolution [2, 6, 60], in the sense that any (non-deterministic) CDCL run on an unsatisfiable formula can be efficiently translated into a resolution refutation of the formula and vice versa. Further results are known for practical CDCL [68] and relations between QBF solving and related proof systems [10, 46]. This allows to apply the plethora of proof complexity results e.g. for propositional and QBF resolution [9, 53, 62] to the analysis of solvers. For example any lower bound for proof size in propositional resolution directly translates into a lower bound for CDCL runtime.

Secondly, proof systems can be employed for *proof logging and certifying solver correctness* by designing certified tools. Therefore, formal proof systems are introduced where a practical proof can be efficiently verified by a relatively simple method and easily emitted during solving [42, 43, 71]. Different proof systems and formats have been designed including RUP, RAT, DRAT and LRAT [27, 38, 39, 44, 70] for SAT and QRAT [45] for QBF. These proof systems have been intensively studied and compared in terms of simulations, e.g., [24, 49, 50]. While the first modelling aspect needs weaker proof systems close to actual solving, the second proof-logging aspect favours very strong proof systems.

In comparison to the rich and intensely researched interplay between solving and proof complexity in SAT and QBF, significantly less is known in this regard for #SAT. In the past five years, *four different #SAT proof systems* have been introduced. These systems are $\textsf{kcps}$ (2019) [21, 22], $\textsf{MICE}$ (2022) [12, 36], $\textsf{CPOG}$ (2023) [18] and most recently $\textsf{CLIP}$ (2024) [23]. These are the only #SAT proof systems so far.

These proof systems are conceptually quite different: while $\textsf{kcps}$ and $\textsf{CPOG}$ are both static proof systems building on circuit classes like Decision-DNNF s used in knowledge compilation [28, 31] on which model counting is efficient, $\textsf{MICE}$ is a rule-based proof system using three simple rules to compute counts for successively more complex formulas. The historically first system $\textsf{kcps}$ was inspired by #SAT solving using knowledge compilation techniques. Both subsequent systems $\textsf{MICE}$ and $\textsf{CPOG}$ were designed with a view towards certifying different #SAT solving approaches. $\textsf{CLIP}$, on the other hand, is a theoretically motivated and very strong proof system similar to implicit proofs [54].

In contrast to the rich proof complexity results for SAT and QBF, not much is known theoretically for these #SAT proof systems. Only for $\textsf{MICE}$, an exponential proof size lower bound was shown last year [12], and it is known that $\textsf{CLIP}$ is the strongest of these systems simulating all others [23]. The relations between the three other systems in terms of simulations have been shown in our conference paper [13] that we extend in this paper.

**Fig. 1:**
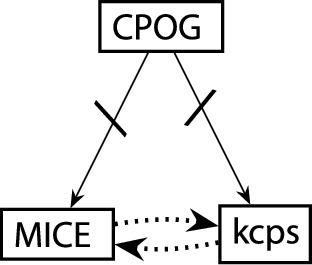
Simulation order of $\textsf{CPOG}$, $\textsf{MICE}$ and $\textsf{kcps}$. A solid crossed edge from *A* to *B* indicates that *A* p-simulates *B* and *A* is exponentially stronger than *B*. Dotted lines indicate incomparability

### Our Contributions

We perform a proof-complexity analysis of the three proof systems $\textsf{kcps}$, $\textsf{MICE}$ and $\textsf{CPOG}$ and completely determine their relative strength in terms of simulations and separations, leading to the picture in Fig. 1. In more detail, our findings can be summarised as follows.

**A. Simulations Between #SAT Proof Systems.** We formally compare the three #SAT proof systems in terms of *simulations* and show that $\textsf{CPOG}$ p-simulates $\textsf{kcps}$ and $\textsf{MICE}$, i.e., both $\textsf{kcps}$ and $\textsf{MICE}$ proofs can be efficiently translated into $\textsf{CPOG}$ proofs.

Rather than showing the two simulations directly, we consider two intermediate proof systems $\mathsf {{kcps}}^\textsf {{Res}}$ and $\mathsf {CPOG^{Decision\text {-}DNNF}}$. The first of these was already suggested as a natural extension of $\textsf{kcps}$ [21], while $\mathsf {CPOG^{Decision\text {-}DNNF}}$ is newly introduced as a restriction of $\textsf{CPOG}$. The system $\textsf{CPOG}$ uses $\textsf{POG}$ s (partitioned-operation graphs) as the underlying circuit class, which in $\mathsf {CPOG^{Decision\text {-}DNNF}}$ is restricted to Decision-DNNF s: the circuit class on which $\textsf{kcps}$ and $\mathsf {{kcps}}^\textsf {{Res}}$ are based. Representing a CNF by any of these circuit models allows efficient counting. Yet, the proofs need to contain additional information as verifying the equivalence of a CNF to a circuit in these models is non-trivial. While the two additional systems simplify our analysis, we believe they are also natural and of independent interest for further research (cf. the discussion in the conclusion).

**Fig. 2:**
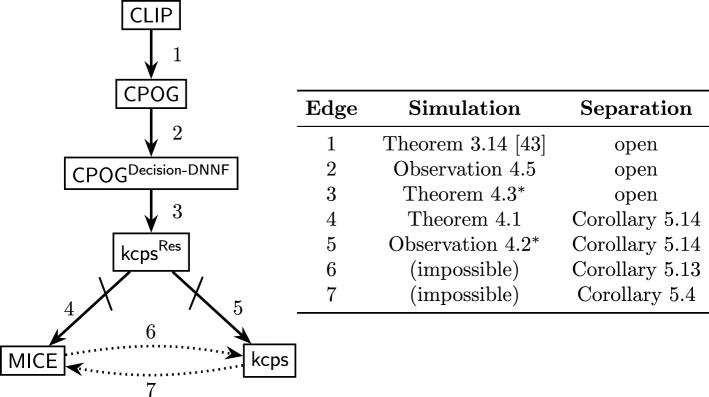
Detailed simulation order of #SAT proof systems. A solid edge from *A* to *B* indicates that *A* p-simulates *B*. If the edge is crossed, *A* is also exponentially separated from *B*. A dotted edge from *A* to *B* indicates that *A* is exponentially separated from *B*. All the simulations of this paper require only logarithmic space; those highlighted with “$*$” only need linear time

For these five proof systems, we determine the simulation order as depicted in Fig. 2, refining Fig. 1 and including pointers to the results. To provide the complete picture, we also add $\textsf{CLIP}$ into the simulation order which is already known to be at least as strong as $\textsf{CPOG}$ [23]. While the simulations of $\textsf{kcps}$ by $\mathsf {{kcps}}^\textsf {{Res}}$ and of $\mathsf {CPOG^{Decision\text {-}DNNF}}$ by $\textsf{CPOG}$ follow almost by definition, the simulations of $\textsf{MICE}$ by $\mathsf {{kcps}}^\textsf {{Res}}$ and $\mathsf {{kcps}}^\textsf {{Res}}$ by $\mathsf {CPOG^{Decision\text {-}DNNF}}$ are more involved—in particular the first one—as they connect conceptually quite different proof formats. The proof systems $\textsf{kcps}$, $\mathsf {{kcps}}^\textsf {{Res}}$, $\mathsf {CPOG^{Decision\text {-}DNNF}}$ and $\textsf{CPOG}$ are all *static* as they are based on circuits equipped with additional information. In contrast, $\textsf{MICE}$ is *rule-based* without any explicit connection to circuits.^1^

**B. Exponential Separations Between #SAT Proof Systems.** As our second main result we establish exponential separations between $\textsf{MICE}$ and $\textsf{kcps}$ in both directions. This entails exhibiting suitable CNF families that have short proofs in $\textsf{MICE}$, while requiring long $\textsf{kcps}$ proofs, and vice versa. As a consequence, both systems are incomparable and at the same time exponentially weaker than $\mathsf {{kcps}}^\textsf {{Res}}$ and $\textsf{CPOG}$, thus resulting in the situation depicted in Fig. 1.

Technically, we obtain one direction ($\textsf{kcps}$ does not simulate $\textsf{MICE}$) by showing a tight characterisation of $\textsf{kcps}$ proof size by regular resolution size on unsatisfiable formulas. A similar characterisation of $\textsf{MICE}$ by full resolution has already been established [12]. As regular resolution is known to be exponentially weaker than resolution [1, 69], the separation follows.

For the other direction ($\textsf{MICE}$ does not simulate $\textsf{kcps}$) we use a variant of the pebbling formulas, prominent in propositional proof complexity [8, 17]. While the small Decision-DNNF s and short $\textsf{kcps}$ proofs are relatively easy to construct, the hardness argument for $\textsf{MICE}$ is more sophisticated (Theorem 5.7).

The first separation positively answers an open question posed by Capelli [21] to find CNFs with polynomial-size Decision-DNNF s (these can always be extracted from short $\textsf{MICE}$ proofs [12]), but no small $\textsf{kcps}$ proofs. The second separation implies that we cannot efficiently transform Decision-DNNF s into $\textsf{MICE}$ proofs.

### Prior Work

This article is an extended version of a conference paper that appeared in the proceedings of the 27th International Conference on Theory and Applications of Satisfiability Testing (SAT 2024) [13]. We extend the conference version by complete proofs and elaborate running examples and detailed examples for each proof system. Moreover, we include the very recently defined proof system $\textsf{CLIP}$  [23], which appeared after our conference version and where the authors show that $\textsf{CLIP}$ simulates all other systems.

### Related Work

For propositional proof systems, there are extensive studies on simulation and separation, see, e.g., [49, 50]. For recently defined proof systems for propositional model counting, this has been open until now. To the best of our knowledge, our conference paper [13] is the first work in this direction for model counting proof systems. However, for individual proof systems there exist already some proof complexity results [18, 21, 36]. Indeed, there are numerous practical exact model counting systems [33, 34], which are based on different techniques. Among these are decomposition-based approaches [5, 52] with component caching [64], dynamic programming [35], knowledge compilation, e.g., c2d [28], d4 [55], as well as hybrid approaches [37]. Further, there are some theoretical results [20] that predate the introduction of formal proof systems for #SAT. There are also clausal proof systems enriched with XOR reasoning [59]. Very recently, first steps on proof systems for approximate counting [58] have been presented.

### Organisation

The remainder of this paper is organised as follows. After reviewing some standard notions from propositional logic and proof systems in Sect. 2, we provide formal definitions of the existing proof systems for #SAT in Sect. 3. We show the simulations from Fig. 2 in Sect. 4. The separations are provided in Sect. 5. We conclude in Sect. 6 with a discussion on practical and theoretical implications.

We highlight that though we believe our results bear practical relevance, this paper performs a purely theoretical proof-complexity investigation.

## Preliminaries

We briefly provide formal notions from propositional logic and proof systems. For more detailed information, we refer to [15, 51]. For an integer *n*, we set $[n] :=\{1, 2, \ldots , n\}$.

*Propositional formulas* A *literal* *l* is a variable *z* or its negation $\overline{z}$, and we write $\textsf{var}(l):= z$. A *clause* is a disjunction of literals, a *conjunctive normal form (CNF)* is a conjunction of clauses. A clause containing only a single literal is called *unit*. Often, we write clauses as sets of literals and formulas as sets of clauses. We assume that *propositional formulas* are given in CNF. We can efficiently transform any formula into a CNF using Tseitin transformations [66]. We want to emphasise that this transformation introduces new auxiliary variables. Although this is harmless for the SAT problem, it may cause complications in the context of model counting. However, as these specific auxiliary variables do not change the model count to the formula, this transformation does not change the complexity of the problem [57].

For a formula *F*, $\textsf{vars}(F)$ denotes the set of all variables in *F*. If $C \in F$ is a clause and $V \subseteq \textsf{vars}(F)$ is a set of variables, we define $C|_V = \{l \in C \mid \textsf{var}(l) \in V\}$ and $F|_V$ denotes the formula *F* with every clause *C* replaced by $C|_V$. $\overline{C}$ denotes the conjunction of all literals $\overline{l}$ for $l \in C$ which we can also interpret as assignment.

Given a set *V* of variables, a (partial) assignment is a (partial) function $\alpha : V \rightarrow \{0,1\}$ that maps variables to Boolean values. We write $\langle V \rangle$ for the set of all $2^{|V |}$ total assignments to *V*. For a (partial) assignment $\alpha$, $F[\alpha ]$ denotes the formula where we replace all occurrences of variables *x* with $\alpha (x)$. If $F[\alpha ] = 1$, we say $\alpha$ *satisfies* *F* and write $\alpha \models F$.

We say that $\alpha$ *falsifies* *F* if $F[\alpha ]=0$ and write $\alpha \not \models F$.

Occasionally, we interpret an assignment as a CNF consisting of precisely those unit clauses that specify the assignment. Therefore, the set operations are well defined for formulas and assignments. We say that two assignments are *consistent* if they assign the same value to all variables in the intersection of their domains.

A formula *F* is *satisfiable* if there exists an assignment $\alpha \in \langle \textsf{vars}(F) \rangle$ such that $\alpha \models F$ and is *unsatisfiable* if there exists no such assignment. For a formula $\varphi$, $\textsf{Mod}(\varphi ) := \{\alpha \in \langle \textsf{vars}(\varphi ) \rangle \mid \alpha \models ~\varphi \}$ is the set of *all models* of $\varphi$. The *model counting problem* (#SAT) asks to compute $|\textsf{Mod}(\varphi )|$ for a given formula $\varphi$. Throughout the paper, we use $\varphi$ for formulas we want to count on. The SAT problem asks to decide whether a given formula is satisfiable and UNSAT whether a given formula is unsatisfiable. Moreover, we need the definition of *semantic consequence*. For a second formula *G*, we write $F \models G$ if and only if for every assignment $\alpha \in \langle \textsf{vars}(F) \rangle$, we have that $\alpha \models F$ implies $\alpha \models G$. We write $F \equiv G$ if and only if $F \models G$ and $G \models F$.

*Proof systems* Following Cook and Reckhow [26], a *proof system* for a language *L* is a polynomial-time computable function *f* with range $\textsf{rng}(f) = L$. Here, *L* will be chosen as either UNSAT or #SAT. If $f(w) = x$, then *w* is called *f*-proof of $x \in L$. In order to compare proof systems we need the notion of *simulations*. Let *P* and *Q* be proof systems for the same language. Then, *P* *p-simulates* *Q* if every *Q*-proof can be translated in polynomial time into a *P*-proof of the same formula. Two proof systems are *p-equivalent* if they p-simulate each other. Further, *P* is *exponentially separated* from *Q* if there is a family of formulas that have polynomial sized *P*-proofs while any *Q*-proof requires exponential size.

*Resolution* is arguably the most studied proof system for UNSAT. It is a *line-based* proof system where new lines are systematically derived from the existing ones. More precisely, the proof lines in resolution are clauses and there are two rules to derive new clauses: The *resolution rule* allows us to derive the clause $C_1 \cup C_2$ from previously derived clauses $C_1 \cup \{x\}$ and $C_2 \cup \{ \overline{x}\}$. With the *weakening rule* we can derive $C_1 \cup C_2$ from a clause $C_1$. A *resolution refutation* of a CNF is a derivation of the empty clause, which we denote as $\square$. As refutational systems, resolution with and without weakening are p-equivalent. Occasionally, when we have to provide resolution proofs we write the resolution step from above as

$$\begin{aligned} {\dfrac{C_1 \cup \{x\} \quad C_2 \cup \{ \overline{x}\}}{C_1 \cup C_2}}. \end{aligned}$$

We can build a whole graph like that where the clauses on top are the clauses from our input formula and the bottom clause is the clause we derive. Thus, we can interpret any proof $\pi$ in a line-based proof system as a directed graph $G_\pi$. For some introduction to graph theory, we refer to the standard literature  [16]. The nodes in $G_\pi$ are proof lines from $\pi$. There is an edge from proof line $l_1$ to $l_2$ if $l_2$ was used to derive $l_1$. A resolution refutation is *regular* if there is no path from the root to a leaf in $G_\pi$ where a variable is resolved more than once.

*Knowledge compilation* As some #SAT proof systems heavily use concepts from knowledge compilation, we start with relevant definitions following standard texts [31, 47]. A *circuit* is a directed acyclic graph with labelled nodes that we call *gates*. We only consider circuits that have exactly one gate with indegree 0. It is called *root* and represents the circuit’s output. Gates with outdegree 0 are called *leaves* and are labelled with literals or constants 0 and 1. The latter are also called *0-gate* or *1-gate*. Every inner gate is an And-, Or- or Not-gate labelled with the corresponding Boolean function. The semantics of such circuits are defined in the usual way. Further, we assume that And- and Or-gates always have exactly two children. Note that this leads to an at most quadratic increase in the size of the circuit.

Let *D* be a circuit. For gates in *D* we use uppercase letters such as *N*. We write $\textsf{vars}(D)$ for the set of all variables occurring in leaves of *D*. $\mathcal {E}(D)$ denotes the Tseitin encoding [66] of *D*, where we use a new variable $\vartheta _{N}$ for every gate *N*. We denote the subcircuit of *D* with root *N* consisting of all descendants of *N* by *D*(*N*).

A circuit is in *negation normal form (NNF)* if it does not contain Not-gates. An And-gate with children $N_1$ and $N_2$ is called *decomposable*, if $\textsf{vars}(D(N_1)) \cap \textsf{vars}(D(N_2)) = \emptyset$. An Or-gate with children $N_1$ and $N_2$ is called *deterministic* if there is no assignment that satisfies both $D(N_1)$ and $D(N_2)$. A DNNF [29] is an NNF where every And-gate is decomposable. A d-DNNF [30] is a DNNF where every Or-gate is deterministic.

**Fig. 3:**
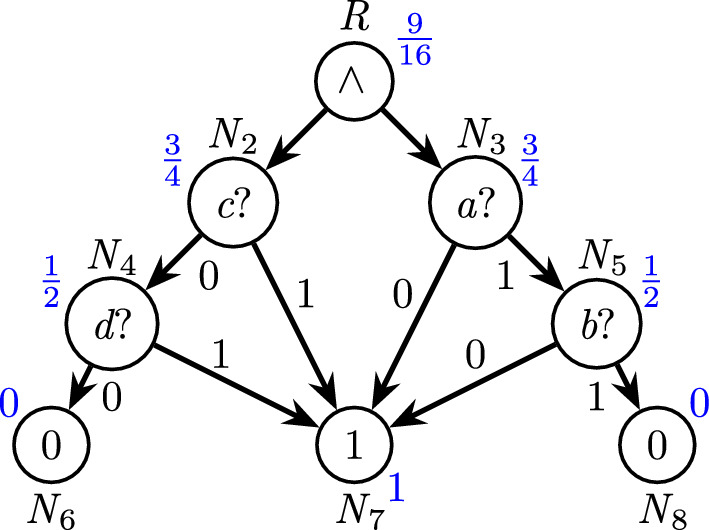
A Decision-DNNF that is equivalent to the formula $\varphi = (\overline{a} \vee \overline{b}) \wedge (c \vee d)$. The blue number at a gate *N* indicates the fraction of assignments that satisfy the subcircuit with root *N*. These numbers are computed bottom-up, i.e., $0$-gate s get count 0, $1$-gate s count 1 and gates that are labelled with a literal count $\frac{1}{2}$. Decision-gate s get the average of the two children, Or-gate s the sum and And-gate s the product. Finally, to compute the model count of $\varphi$, we multiply the fraction of satisfying assignments of the whole Decision-DNNF with the count of all possible assignments to $\textsf{vars}(\varphi )$, resulting in $|\textsf{Mod}(\varphi ) | = \frac{9}{16} \cdot 2^{|\textsf{vars}(\varphi ) |} = 9$

Since it is non-trivial to check if all Or-gate s are indeed deterministic, we also consider a restricted version of d-DNNF called Decision-DNNF. In a Decision-DNNF, any Or-gate has the form $N = (N_1 \texttt { or } N_2)$ with $N_1 = (x \texttt { and } N_3)$ and $N_2 = (\overline{x} \texttt { and } N_4)$ for any variable *x*. It is obvious that any such Or-gate is deterministic. For better readability, we write Decision-DNNFs without Or-gate s but use Decision-gate s instead. We rewrite the above gate as $N = (\texttt {if }x\texttt { then }N_3\texttt { else }N_4)$. Note that we can assume that the leaves of a Decision-DNNF contain only constants 0 or 1. Figure 3 displays a Decision-DNNF.

Further, in any path from the root to a leaf, any variable can be decided at most once because of the decomposability property. We say that an assignment $\alpha$ *reaches* a gate *N* if there is a path *P* from the root to *N* such that all decisions along *P* are consistent with $\alpha$. As such circuits represent formulas, we write $\varphi \equiv D$ to notate that the formula corresponding to circuit *D* is semantically equivalent to a CNF $\varphi$.

## Proof Systems for #SAT

In this section, we recall the existing #SAT proof systems $\textsf{kcps}$, $\textsf{CPOG}$, $\textsf{MICE}$, and $\textsf{CLIP}$ and provide some intuition. In particular, we provide a concise formalisation of $\textsf{CPOG}$. Furthermore, we introduce two adapted versions of $\textsf{kcps}$ and $\textsf{CPOG}$ that we call $\mathsf {{kcps}}^\textsf {{Res}}$ and $\mathsf {CPOG^{Decision\text {-}DNNF}}$. For some better understanding and intuition we provide a proof for the very simple formula $\varphi = (\overline{a} \vee \overline{b}) \wedge (c \vee d)$ in each proof system.

### kcps: knowledge Compilation Proof System

The system $\textsf{kcps}$ is the historically first proof system for #SAT, introduced by Capelli in 2019  [21]. Note that there is also a similar system [22] which we do not consider in this paper since its proof complexity is already analysed [14]. As the name suggests, it aims to certify solvers that apply knowledge compilation techniques. These solvers transform the input CNF into a format that can handle various queries efficiently, in particular model counting [28, 31, 55]. As in practice solvers often rely on compiling the formulas into Decision-DNNF s, $\textsf{kcps}$ is based on this class of circuits.

A $\textsf{kcps}$ proof of $\varphi$ provides a Decision-DNNF  *D* such that $D \equiv \varphi$. The Decision-DNNF  *D* implicitly contains the model count of $\varphi$ as we can efficiently compute it [31] (cf. Fig 3 for an example). However, for this to be a proof in the sense of Cook-Reckhow [26], we need to verify that *D* and $\varphi$ are indeed equivalent. The direction $D \models \varphi$ can always be checked efficiently [31], Lemma 3.10 below provides for a formal argument for that. However, the other direction $\varphi \models D$ is a coNP-complete problem for arbitrary Decision-DNNF s [21]. Thus, we consider a restricted version of Decision-DNNF s on which checking $\varphi \models D$ becomes easy as well. For that, we review the notion of a certified Decision-DNNF  [21].

#### Definition 3.1

(*S*-certified Decision-DNNF [21]) Let *S* be a set of clauses and *D* a Decision-DNNF. A clause is a *certificate* for a $0$-gate  $N \in D$ if all assignments that reach *N* falsify *C*. *D* is called *S-certified* if every 0-gate is labelled by a certificate $C \in S$.

These restricted Decision-DNNF s have the property that for a formula $\varphi$, any $\varphi$-certified Decision-DNNF  *D* satisfies $\varphi \models D$ [21]. To see this, consider the equivalent statement $\lnot D \models \lnot \varphi$. Let $\alpha$ be an assignment that falsifies *D*, then it reaches a $0$-gate. Consequently, it has to falsify its certificate and in particular $\varphi$.

Finally, we can define the $\textsf{kcps}$ proof system:

#### Definition 3.2

($\textsf{kcps}$ [21]) A $\textsf{kcps}$ proof of a CNF $\varphi$ is a $\varphi$-certified Decision-DNNF *D* where $D \equiv \varphi$.

Note that the model count of $\varphi$ and also the equivalence between $\varphi$ and *D* are not explicitly part of the proof as we can compute the model count from *D* and verify $D \equiv \varphi$, both in polynomial time.

#### Example 3.3

For a $\textsf{kcps}$ proof of $\varphi = (\overline{a} \vee \overline{b}) \wedge (c \vee d)$ we can choose the Decision-DNNF *D* from Fig 3, where we additionally label both $0$-gate s with some clause $C \in \varphi$. For the left $0$-gate we can use the certificate $C = (c \vee d)$, as every path from the root to this gate assigns $c=0$ and $d=0$ and thus falsifies *C*. Note that in this simple example there is only one such path, but it would be possible that there are several paths. Similarly, we can use the certificate $C= (\overline{a} \vee \overline{b})$ for the right $0$-gate.

With these labels, it is ensured that $\varphi \models D$, the other direction, $D \models \varphi$, is not explicitly part of the proof, but has to be satisfied as well. For that, assume that $\alpha \in \langle \{a,b,c,d\} \rangle$ satisfies *D*, i.e., both paths from *R* land in the $1$-gate. Thus, *c* or *d* have to be assigned to 1 and *a* or *b* to 0 implying that $\alpha$ satisfied $\varphi$.

Therefore, when we label all $0$-gate s accordingly, we obtain a $\varphi$-certified Decision-DNNF which is a valid $\textsf{kcps}$ proof for $\varphi$.

In fact, Capelli [21] proposed a generalisation of $\textsf{kcps}$ where the certifying clauses for the $0$-gate s are not necessarily clauses of the original formula $\varphi$. Instead, we use as certificates arbitrary clauses derived by resolution from $\varphi$. This results in the proof system $\mathsf {{kcps}}^\textsf {{Res}}$.

#### Definition 3.4

($\mathsf {{kcps}}^\textsf {{Res}}$) A $\mathsf {{kcps}}^\textsf {{Res}}$ proof of a CNF $\varphi$ is a pair $(\sigma , D)$ where

1. $\sigma$ is a resolution derivation starting from the clauses in $\varphi$ and
2. *D* is a $\sigma$-certified Decision-DNNF (i.e., all clauses labelling the $0$-gate s in *D* are derived in $\sigma$) such that $D \equiv \varphi$.

#### Example 3.5

As $\mathsf {{kcps}}^\textsf {{Res}}$ is a generalisation of $\textsf{kcps}$, the $\textsf{kcps}$ proof from Example 3.3 is also a valid $\mathsf {{kcps}}^\textsf {{Res}}$ proof by adding $\sigma$ only containing both clauses of $\varphi$.

To illustrate the difference to $\textsf{kcps}$, let us consider the formula $\psi = (\overline{a} \vee \overline{b} \vee c) \wedge (\overline{a} \vee \overline{b} \vee \overline{c}) \wedge (c \vee d)$. It is obvious, that $\varphi \equiv \psi$ and thus $D \equiv \psi$ for the Decision-DNNF from Fig 3. However, we cannot use *D* for a $\textsf{kcps}$ proof as the right $0$-gate cannot be certified by a clause $C \in \psi$. Nonetheless, we can derive the clause $C = (\overline{a} \vee \overline{b})$ in $\sigma$ with a single resolution step and use $C \in \sigma$ as a certificate leading to a valid $\mathsf {{kcps}}^\textsf {{Res}}$ proof of $\psi$.

Furthermore, we want to highlight that for any unsatisfiable formula $\Phi$, a $\mathsf {{kcps}}^\textsf {{Res}}$ proof can use a Decision-DNNF with a single $0$-gate that is labelled with the empty clause which we derive from $\Phi$ with resolution.

Finally, we want to point out, that we cannot take arbitrary Decision-DNNF $D \equiv \varphi$ for $\mathsf {{kcps}}^\textsf {{Res}}$ proofs as there might be a $0$-gate for which no certificate exists. To illustrate this, assume, that we would *merge* the two $0$-gate s of the Decision-DNNF from Fig 3. There would be two paths from the root to the $0$-gate, one assigns $c=0$ and $d=0$ while the other assigns $a = 1$ and $b=1$. Thus, we cannot find any clause *C* that is entailed by $\varphi$ and could be used as certificate.

### CPOG: Certified Partitioned-Operation Graphs

In contrast to $\textsf{kcps}$, $\textsf{CPOG}$ is not restricted to certified Decision-DNNF s, but uses the more flexible circuit class $\textsf{POG}$ (partitioned-operation graphs). Instead of providing the original definition of $\textsf{POG}$ s from [18], we equivalently define a $\textsf{POG}$ as a d-DNNF with Not-gate s (alternatively, a d-DNNF can be viewed as a POG with negation applied only to variables).

**Fig. 4:**
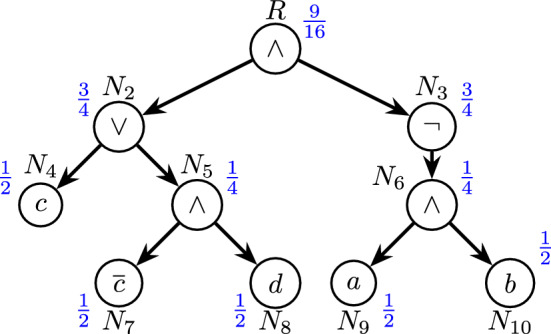
A $\textsf{POG}$ that is equivalent to the formula $\varphi = (\overline{a} \vee \overline{b}) \wedge (c \vee d)$. The blue number at a gate *N* indicates the fraction of assignments that satisfy the subcircuit with root *N*. These numbers are computed bottom-up similar to the computation for Decision-DNNF s illustrated in Fig 3. Leaves labelled with a literal get count $\frac{1}{2}$, Or-gate s get the average of the two children, and And-gate s the product. Finally, to compute the model count of $\varphi$, we multiply the fraction of satisfying assignments of the whole $\textsf{POG}$ with the count of all possible assignments to $\textsf{vars}(\varphi )$, resulting in $|\textsf{Mod}(\varphi ) | = \frac{9}{16} \cdot 2^{|\textsf{vars}(\varphi ) |} = 9$

Model counting is also efficient on $\textsf{POG}$ s [18], and in fact $\textsf{POG}$ s appear to be the largest class to which the model counting idea used for Decision-DNNF s naturally extends. Fig 4 illustrates a $\textsf{POG}$ and how to count on it. In order to maintain efficient proof checking, a $\textsf{CPOG}$ proof has to explicitly prove that *P* is indeed a $\textsf{POG}$ and that $\varphi \equiv P$. This leads to the following definition.

#### Definition 3.6

($\textsf{CPOG}$ [18]) A $\textsf{CPOG}$ proof of a CNF $\varphi$ is a 4-tuple $(\mathcal {E}(P), \rho , \psi , X)$ where

1. *P* is a $\textsf{POG}$ with root *R* such that $\mathcal {E}(P)$ is a clausal encoding of *P* that uses a fresh variable $\vartheta _{N}$ for every gate $N \in P$,
2. $\rho$ is a proof for $\varphi \models P$, i.e., $\rho$ is a resolution refutation of $\varphi \wedge \mathcal {E}(P) \wedge (\overline{\vartheta _{R}})$,
3. $\psi$ is a proof for $P\models \varphi$, i.e., $\psi$ contains a resolution refutation of $\mathcal {E}(P) \wedge (\vartheta _{R}) \wedge \overline{C}$ for every clause $C \in \varphi$,
4. *X* is a set of proofs verifying that all Or-gate s of *P* are deterministic, i.e., *X* is a set of resolution refutations such that for any Or-gate *N*, *X* contains a resolution refutation of $\mathcal {E}(P) \wedge (\vartheta _{N_1}) \wedge (\vartheta _{N_2})$, where $N_1$ and $N_2$ are the two child gates of *N*.

Note that the main idea of this proof system is that the $\textsf{POG}$ in any valid $\textsf{CPOG}$ proof satisfies $P \equiv \varphi$, which is ensured by Conditions 2 and 3. Further, $\textsf{CPOG}$ is originally defined on circuits with arbitrary fan-in, however we consider only the binary case which is polynomially equivalent. Additionally, the original definition uses RUP steps for the propositional proofs, which are p-equivalent to resolution. In our definition, we use resolution proofs instead.

#### Example 3.7

**Fig. 5:**
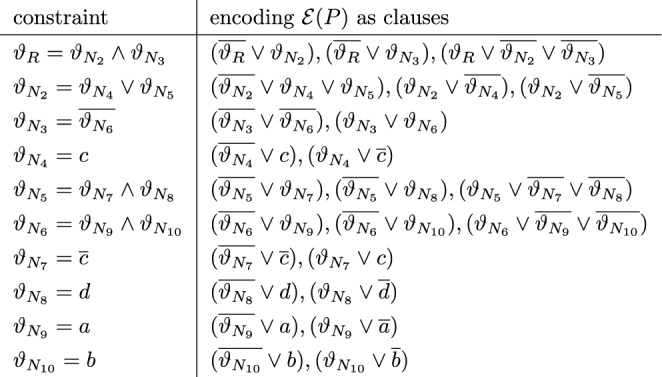
The encoding $\mathcal {E}(P)$ from the $\textsf{POG}$ *P* from Fig. 4

We construct a $\textsf{CPOG}$ proof $(\mathcal {E}(P), \rho , \psi , X)$ for $\varphi = (\overline{a} \vee \overline{b}) \wedge (c \vee d)$ As underlying POG *P*, we use the circuit from Fig 4. Its encoding $\mathcal {E}(P)$ is given in Fig 5.

Next, we have to provide a resolution refutation $\rho$ of $\varphi \wedge \mathcal {E}(P) \wedge (\overline{\vartheta _{R}})$. We show this in three parts.

The clause $(c \vee d)$ enforces $\vartheta _{N_2}$ to be satisfied: 
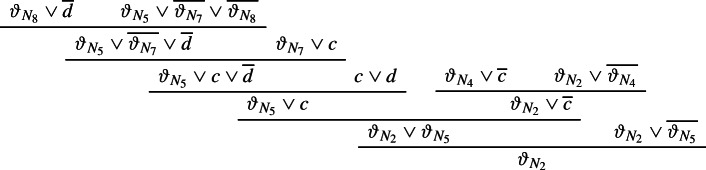
 The clause $(\overline{a} \vee \overline{b})$ enforces $\vartheta _{N_3}$ to be satisfied: 
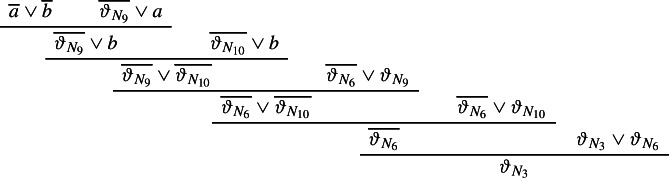
 Then, also $\vartheta _{R}$ is satisfied contradicting $(\overline{\vartheta _{R}})$: 
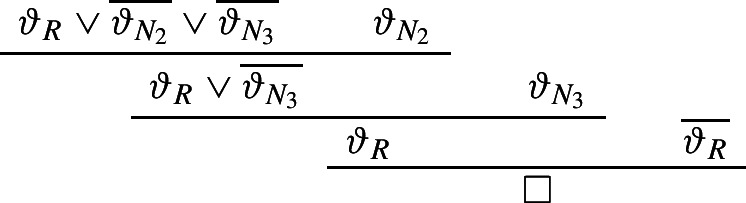


Next, for $\psi$ we need a resolution refutation of $\mathcal {E}(P) \wedge (\vartheta _{R}) \wedge \overline{C}$ for both clauses $C \in \varphi$.

For, $C = (\overline{a} \vee \overline{b})$, we observe that $\overline{C}$ enforces $a = 1$ and $b=1$. Thus, $\vartheta _{N_6} = 1$, $\vartheta _{N_3} = 0$ and $\vartheta _{N_R} = 0$:
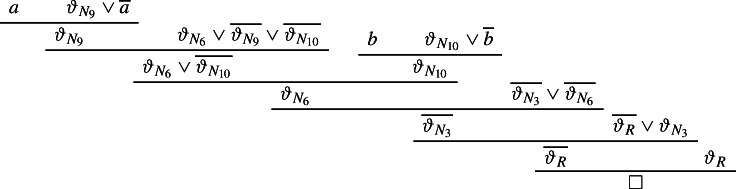
 Similarly, for $C = (c \vee d)$, $\overline{C}$ enforces $c = 0$ and $d=0$. Thus, $\vartheta _{N_5} = 0$, $\vartheta _{N_2} = 0$ and $\vartheta _{N_R} = 0$:
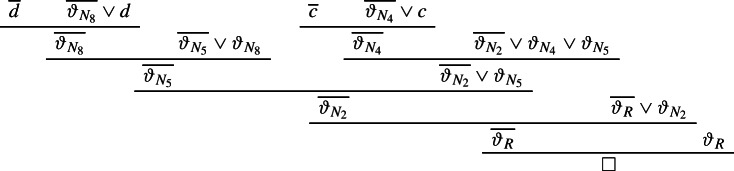


Finally, we need a set *X* of resolution refutations proving that all Or-gate s are indeed deterministic. As $N_2$ is the only Or-gate, we have to refute $\mathcal {E}(P) \wedge (\vartheta _{N_4}) \wedge (\vartheta _{N_5})$. For that, observe that $\vartheta _{N_4}$ enforces *c* while $\vartheta _{N_5}$ enforces $\overline{c}$:
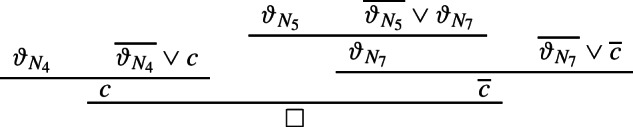


Putting these things together, $(\mathcal {E}(P), \rho , \psi , X)$ is a valid $\textsf{CPOG}$ proof for $\varphi$.

The underlying structure of $\textsf{POG}$ s in $\textsf{CPOG}$ proofs is quite generic. So far, the only implementation of $\textsf{CPOG}$ [18] is restricted to Decision-DNNF s instead of $\textsf{POG}$ s. We capture this variant in the following definition:

#### Definition 3.8

($\mathsf {CPOG^{Decision\text {-}DNNF}}$) A $\mathsf {CPOG^{Decision\text {-}DNNF}}$ proof of a CNF $\varphi$ is a pair $(\mathcal {E}(D), \rho )$ where

1. *D* is a Decision-DNNF with root *R* such that $\mathcal {E}(D)$ is a clausal encoding of *D* that uses a fresh variable $\vartheta _{N}$ for every gate $N \in D$, and $D \models \varphi$,
2. $\rho$ is a proof for $\varphi \models D$, i.e., $\rho$ is a resolution refutation of $\varphi \wedge \mathcal {E}(D) \wedge (\overline{\vartheta _{R}})$.

#### Example 3.9

**Fig. 6:**
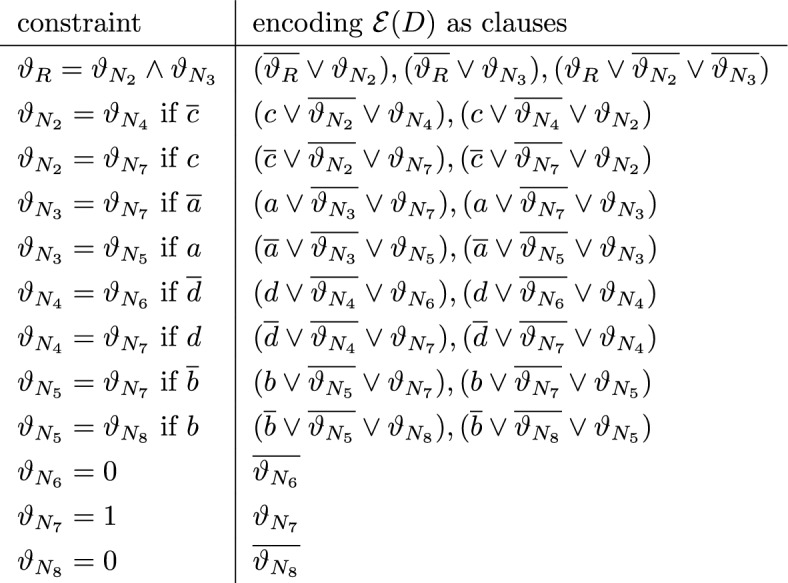
The encoding $\mathcal {E}(D)$ from the Decision-DNNF *D* from Fig. 3

In order to prove $\varphi = (\overline{a} \vee \overline{b}) \wedge (c \vee d)$ with $\mathsf {CPOG^{Decision\text {-}DNNF}}$, we use the Decision-DNNF *D* from Fig. 3. The corresponding encoding $\mathcal {E}(D)$ is given in Fig. 6. Next, we need a resolution refutation $\rho$ of $\varphi \wedge \mathcal {E}(D) \wedge (\overline{\vartheta _{R}})$.

For that, we first show that clause $(c \vee d)$ enforces $\vartheta _{N_2}$ to be true: 
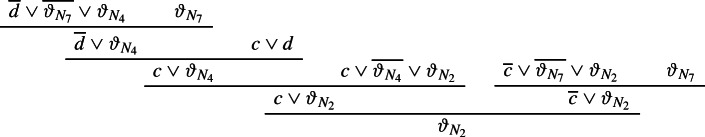
 Next, we show that $(\overline{a} \vee \overline{b})$ enforces $\vartheta _{N_2}$ to be true:
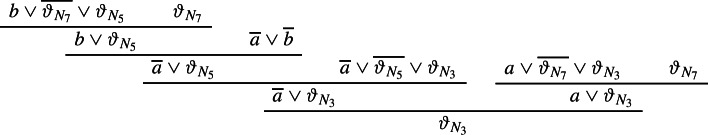
 With that, we finally get the contradiction as follows:
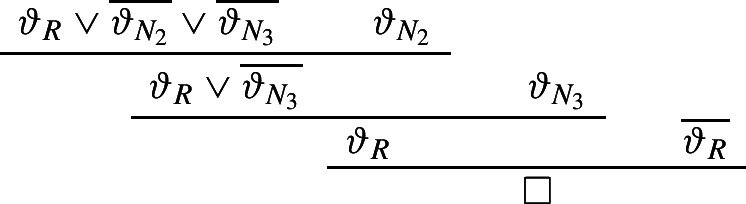
 This resolution refutation proves that $\varphi \models D$. The other direction $D \models \varphi$ holds as well, as we argued in Example 3.3. However, this direction is not explicitly part of the proof in contrast to a $\textsf{CPOG}$ proof. Thus, the $\mathsf {CPOG^{Decision\text {-}DNNF}}$ proof for $\varphi$ is finished.

We see in Example 3.9 that in comparison to $\textsf{CPOG}$ (Definition 3.6), for $\mathsf {CPOG^{Decision\text {-}DNNF}}$ (Definition 3.8) the last two items are missing. This is clear for Condition 4 as *D* only contains Decision-gate s instead of Or-gate s. As the Decision-DNNF should satisfy $D \equiv \varphi$, we have to require $D\models \varphi$. However, we do not have to provide any proof of that, because the proof $\psi$ in Condition 3 can always be computed efficiently for Decision-DNNF s as we show in the next lemma:

#### Lemma 3.10

Let *D* be a Decision-DNNF with root *R* and encoding $\mathcal {E}(D)$. If $D \equiv \varphi$, then we can compute $\psi$, i.e., a resolution refutation of $\mathcal {E}(D) \wedge (\vartheta _{R}) \wedge \overline{C}$ for every clause $C \in \varphi$, in time $O(|D | \cdot |\varphi |)$.

#### Proof

Let $C \in \varphi$ be some arbitrary fixed clause. We assume that $D \equiv \varphi$. Let $L = N_1, \dots , N_n$ be a list of all gates in *D* that are unsatisfiable under the partial assignment $\overline{C}$ in some topological ordering such that no gate is listed after its ancestors. Note that *R* is the last element in *L* as $D[\overline{C}] \equiv \varphi [\overline{C}]$ has to be unsatisfiable.

We show inductively that for every $i \in [n]$ we can effectively derive the unit clause $(\overline{\vartheta _{N_i}})$ from $\mathcal {E}(D) \wedge \overline{C}$. For that, we assume that we have already derived all clauses $(\overline{\vartheta _{N_{i}}})$ for $i \in [k-1]$. In order to derive $(\overline{\vartheta _{N_{k}}})$, we distinguish what kind of gate $N_k$ is.

$N_k$ is a leaf. Since $N_k \in L$, $N_k$ cannot be an $1$-gate. If $N_k$ is a $0$-gate, then we do not have to derive anything because $(\overline{\vartheta _{N_k}}) \in \mathcal {E}(D)$.

$N_k$ *is an* And-gate. Let $N^1$ and $N^2$ be the children of $N_k$. Since $N_k \in L$, at least one of the children has to be in *L* as well, we assume w.l.o.g. $N^1 \in L$. Because of the ordering of *L*, we have already derived $(\overline{\vartheta _{N^1}})$ per induction hypothesis. Further, there has to be a clause $(\vartheta _{N^1} \vee \overline{\vartheta _{N_k}}) \in \mathcal {E}(D)$. By resolving with $(\overline{\vartheta _{N^1}})$ we obtain the clause $(\overline{\vartheta _{N_k}})$.

$N_k$ *is a* Decision-gate. Let *x* be the variable that is decided by $N_k$. Let $N_k$ be a Decision-gate deciding some variable *v*. Further, let $N^0$ be the child for the case $v = 0$ and $N^1$ the child for $v=1$. Then, this Decision-gate $N_k = (\texttt {if }v\texttt { then }N^1\texttt { else }N^0)$ is the simpler notation for $N_k = (N^2 \texttt { or } N^3)$ with $N^2 = (N^4 \texttt { and } N^1)$, $N^4 = (v)$, $N^3 = (N^5 \texttt { and } N^0)$, and $N^5 = (\overline{v})$. In the following we use that $\mathcal {E}(D)$ contains the clauses $(v \vee \overline{\vartheta _{N^5}})$, $(\vartheta _{N^5} \vee \overline{\vartheta _{N^3}})$, $(\vartheta _{N^1} \vee \overline{\vartheta _{N^3}})$, $(\vartheta _{N^0} \vee \overline{\vartheta _{N^2}})$, and $(\vartheta _{N^2} \vee \vartheta _{N^3} \vee \overline{\vartheta _{N_k}})$.

- *Case 1.* We assume w.l.o.g. that the literal *v* occurs in *C* (the case that $\overline{v}$ occurs in *C* is symmetrical). Then, we have the unit clause $(\overline{v}) \in \overline{C}$ and $N^0 \in L$, i.e., we have already derived $(\overline{\vartheta _{N^0}})$ per induction hypothesis. We obtain $(\overline{\vartheta _{N_k}})$ with 
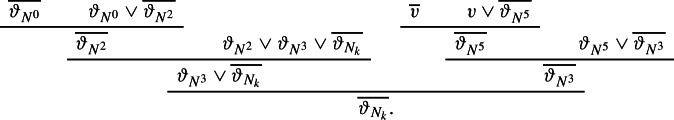
- *Case 2.* The variable *v* does not occur in *C*. Then, both $N^0$ and $N^1$ have to be in *L* as otherwise if one of these two gates would be satisfiable, we could extend the satisfying assignment such that it also satisfies $N_k$. Therefore, we have already derived $(\overline{\vartheta _{N^0}})$ and $(\overline{\vartheta _{N^1}})$ per induction hypothesis. We obtain $(\overline{\vartheta _{N_k}})$ with 
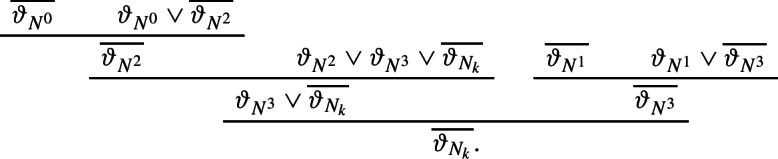

This completes the induction. Since $R \in L$, we can derive $(\overline{\vartheta _{R}})$ efficiently. With an additional resolution step with the unit clause $(\vartheta _{R})$, we derive the empty clause. In total, we have constructed a resolution refutation of $\mathcal {E}(D) \wedge (\vartheta _{R}) \wedge \overline{C}$ of size at most $11 \cdot |L | \le 11 \cdot |D |$. Since $\psi$ contains such a refutation for every clause $C \in \varphi$, we can construct $\psi$ with at most $11 \cdot |D | \cdot |\varphi |$ resolution steps. $\square$

Therefore, $\textsf{CPOG}$ is indeed a generalisation of $\mathsf {CPOG^{Decision\text {-}DNNF}}$. However, it is unknown whether the two systems can be separated. Since $\textsf{POG}$ s are a generalisation of d-DNNF, which are exponentially separated from Decision-DNNF s [7], it follows that $\textsf{POG}$ s are more succinct than Decision-DNNF s. The known separating formulas, however, do not admit short CNF representations. Thus, the question whether $\textsf{CPOG}$ is exponentially stronger than $\mathsf {CPOG^{Decision\text {-}DNNF}}$ remains open.

**Fig. 7:**
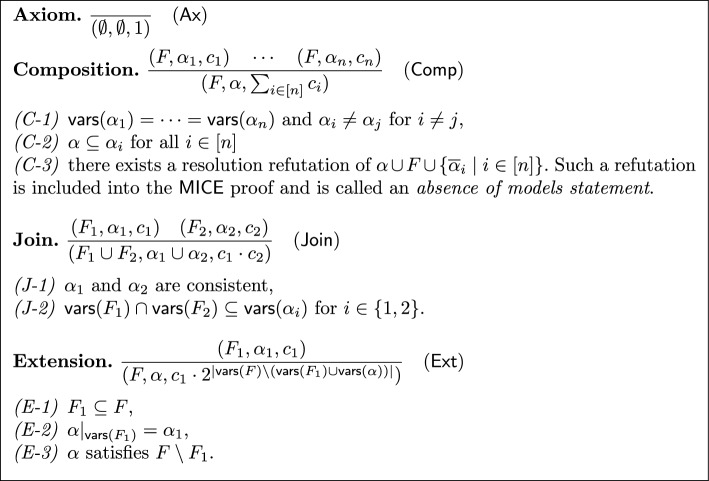
Inference rules for $\textsf{MICE}$ in a simplified version [12]

### MICE: Model-Counting Induction by Claim Extension

The third system we need is the line-based #SAT proof system $\textsf{MICE}$, introduced with the intention to provide a proof system close to various solvers [36]. Here, we use $\textsf{MICE}$ in its slightly simplified, but p-equivalent form as defined by Beyersdorff et al. [12].

#### Definition 3.11

($\textsf{MICE}$ [12, 36]) The proof lines in $\textsf{MICE}$ are called *claims*. A *claim* is a 3-tuple $(F,\alpha ,c)$ consisting of a CNF *F*, a partial assignment $\alpha$ of $\textsf{vars}(F)$ (called *assumption*) and a count *c*. A $\textsf{MICE}$ *proof* of a CNF $\varphi$ is a sequence of claims $I_1, \dots , I_k$ that are derived with the inference rules in Fig. 7 such that the final claim has the form $(\varphi , \emptyset , c)$ for some count *c*.

If a $\textsf{MICE}$ proof $\pi$ derives the claim $(\varphi , \emptyset , c)$, then $\pi$ proves that $\varphi$ has exactly *c* models. A claim $(F,\alpha ,c)$ is *correct* if *F* has exactly *c* models that are consistent with $\alpha$. Since only correct claims can be derived in $\textsf{MICE}$ [12], the count *c* of a correct claim $(F,\alpha ,c)$ is uniquely determined by *F* and $\alpha$. Thus, we sometimes omit *c* and use the notation $(F,\alpha )$ instead.

#### Example 3.12

In order to prove $\varphi = (\overline{a} \vee \overline{b}) \wedge (c \vee d)$ with $\textsf{MICE}$, we use (Ax) to derive claim

$$\begin{aligned} I_1 = (\emptyset , \emptyset , 1). \end{aligned}$$

From that we apply (Ext) three times and obtain claims

$$ ((\overline{a} \vee \overline{b}), \{a=0, b=0\}, 1),\; ((\overline{a} \vee \overline{b}), \{a=0, b=1\}, 1),\; ((\overline{a} \vee \overline{b}), \{a=1, b=0\}, 1). $$

We use (Comp) with these three claims to derive

$$\begin{aligned} I_2 = ((\overline{a} \vee \overline{b}), \emptyset , 3), \end{aligned}$$

where we use the following refutation of $(\overline{a} \vee \overline{b}) \wedge ({a} \vee {b}) \wedge ({a} \vee \overline{b}) \wedge (\overline{a} \vee {b})$ as absence of models statement:
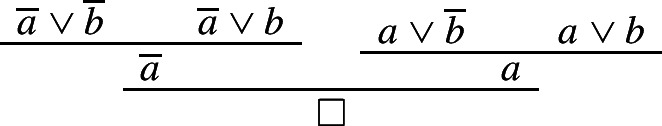
 In a similar fashion, we apply (Ext) again three times to $I_1$ leading to claims:

$$ ((c \vee d), \{c=0, d=1\}, 1), \; ((c \vee d), \{c=1, d=0\}, 1), \; ((c \vee d), \{c=1, d=1\}, 1). $$

We use (Comp) with these three claims to derive

$$\begin{aligned} I_3 = ((c \vee d), \emptyset , 3), \end{aligned}$$

where we use the following refutation of $(c \vee d) \wedge (c \vee \overline{d}) \wedge (\overline{c} \vee d) \wedge (\overline{c} \vee \overline{d})$ as absence of models statement: 
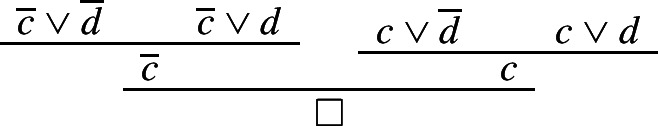
 Finally, we apply (Join) to $I_2$ and $I_3$ leading to

$$\begin{aligned} (\varphi , \emptyset , 9) \end{aligned}$$

finishing the $\textsf{MICE}$ proof.

Note that we can always construct a naive $\textsf{MICE}$ proof for any formula $\varphi$ by considering every model separately in the following way: we start with (Ax) and derive claims $I_\alpha = (\varphi , \alpha , 1)$ for every assignment $\alpha \in \textsf{Mod}(\varphi )$ with (Ext). Afterwards, we combine all these claims with (Comp) to the final claim $(\varphi , \emptyset , |\textsf{Mod}(\varphi ) |)$. The formula $\varphi \cup \{\overline{\alpha } \mid \alpha \in \textsf{Mod}(\varphi )\}$ has to be unsatisfiable and thus can be refuted with resolution, i.e. there is always a valid absence of models statement for the (Comp) step. In particular, this construction shows the completeness of the $\textsf{MICE}$ system, even without the (Join) rule.

### CLIP: Circuit Linear Induction Proposition

For a formula $\varphi$, let us fix an ordering of the variables $\textsf{vars}(\varphi )$. This induces a lexicographic ordering of the assignments $\langle \textsf{vars}(\varphi ) \rangle$. Using this ordering, we write $\alpha \le \beta$ if $\alpha$ precedes $\beta$ or $\alpha = \beta$, and $\beta = \alpha + 1$ if $\beta$ is the immediate successor of $\alpha$. Furthermore, let $\textbf{0}$ and $\textbf{1}$ denote the assignments setting all variables to 0 or 1, respectively. The cumulative count up to an assignment $\alpha$, called $c(\varphi , \alpha )$, is defined as

$$\begin{aligned} c(\varphi , \alpha ) = |\{\beta \in \langle \textsf{vars}(\varphi ) \rangle \mid \beta \le \alpha \text { and } \beta \models \varphi \} |. \end{aligned}$$

Next, let *C* be a circuit that takes an assignment $\alpha \in \langle \textsf{vars}(\varphi ) \rangle$ as input, i.e. it has an input gate for every variable $v \in \textsf{vars}(\varphi )$. The output gates of the circuit encode a binary number. We say that *C* computes the cumulative count of $\varphi$ if $C(\alpha ) = c(\varphi , \alpha )$ for all $\alpha \in \langle \textsf{vars}(\varphi ) \rangle$.

A proof for formula $\varphi$ in the $\textsf{CLIP}$ proof system [23] provides such a circuit *C* computing the cumulative counts of $\varphi$ together with a propositional proof that *C* has indeed this property. Note that the model count can be efficiently computed from *C* because of $|\textsf{Mod}(\varphi ) | = C({\textbf {1}})$. In order to show $C(\alpha ) = c(\varphi , \alpha )$ for all assignments $\alpha \in \langle \textsf{vars}(\varphi ) \rangle$, we use an inductive approach. For that, we encode the following two statements in propositional logic:

$$\begin{aligned} C(\textbf{0})&= {\left\{ \begin{array}{ll} 1 \text { if } \textbf{0} \models \varphi \\ 0 \text { otherwise,} \end{array}\right. }\\ C(\beta )&= C(\alpha ) + {\left\{ \begin{array}{ll} 1 \text { if } \beta \models \varphi \\ 0 \text { otherwise} \end{array}\right. } \end{aligned}$$

for all assignments $\alpha$ and $\beta = \alpha +1$. We prove these two statements using a propositional proof system, such as extended resolution.

**Fig. 8:**
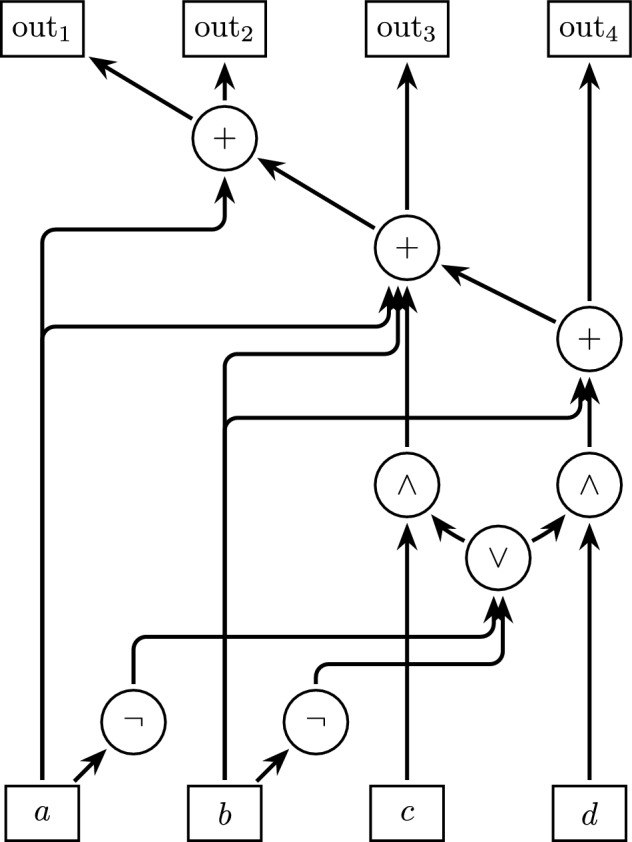
Circuit *C* for a $\textsf{CLIP}$ proof of $\varphi = (\overline{a} \vee \overline{b}) \wedge (c \vee d)$. The output is the binary number $[\text {out}_1 \text {out}_2 \text {out}_3 \text {out}_4]_2$. The addition-nodes (+) are shorthand for adder circuits, which output the sum of their input bits as a two-bit binary number

#### Example 3.13

In order to prove $\varphi = (\overline{a} \vee \overline{b}) \wedge (c \vee d)$ with $\textsf{CLIP}$, we need a circuit that computes the cumulative counts of $\varphi$:
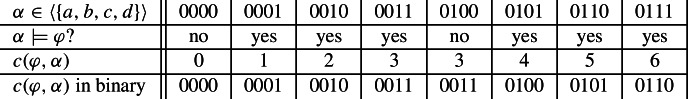

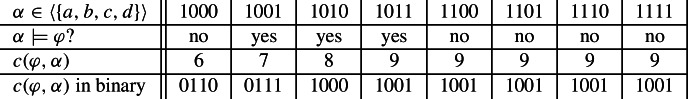


Such a circuit *C* computing the cumulative counts for $\varphi$ is given in Fig. 8. While it is easy to verify that *C* indeed outputs $c(\varphi , \alpha )$ in binary for any input assignment $\alpha \in \langle \{a,b,c,d\} \rangle$, in a $\textsf{CLIP}$ proof, we would need to show the two invariants mentioned above formally with the inductive approach. The correctness of $C(\textbf{0})$ is easy to verify. For the inductive step, we encode the following statement as CNF using a Tseitin encoding:

$$ \psi (\alpha ) = \left( C(\alpha +1) \ne C(\alpha ) + {\left\{ \begin{array}{ll} 1 \text { if } \alpha +1 \models \varphi \\ 0 \text { otherwise} \end{array}\right. } \right) . $$

This CNF is unsatisfiable and can be refuted with any propositional proof system. The $\textsf{CLIP}$ proof contains such a refutation, which we omit for brevity.

$\textsf{CLIP}$ is a very powerful system, which is illustrated by the following result:

#### Theorem 3.14

([23]) $$\textsf{CLIP}$$ p-simulates $$\textsf{CPOG}$$.

This means that any $\textsf{CPOG}$ proof of $\varphi$ can be efficiently transformed into a circuit *C* together with a propositional proof certifying that *C* computes the cumulative counts of $\varphi$.

## CPOG Simulates MICE and Kcps

We start our investigation by clarifying the simulation order of the #SAT proof systems introduced in Sect. 3 and prove that $\textsf{CPOG}$ simulates $\textsf{MICE}$ and $\textsf{kcps}$. We achieve this by efficiently constructing $\textsf{CPOG}$ proofs from given $\textsf{MICE}$ or $\textsf{kcps}$ proofs. We use the systems $\mathsf {CPOG^{Decision\text {-}DNNF}}$ and $\mathsf {{kcps}}^\textsf {{Res}}$ from Sect. 3 as convenient intermediate proof systems and show the four simulations depicted in Fig. 2.

The first simulation transforms $\textsf{MICE}$ proofs into $\mathsf {{kcps}}^\textsf {{Res}}$ proofs:

### Theorem 4.1

$$\mathsf {{kcps}}^\textsf {{Res}}$$ p-simulates $$\textsf{MICE}$$.

### Proof

Let $\pi = I_1, \dots , I_n$ be a $\textsf{MICE}$ proof of a CNF $\varphi$ with $I_k = (F_k,\alpha _k)$ for every $k \in [n]$. Our goal is to construct a $\mathsf {{kcps}}^\textsf {{Res}}$ proof for $\varphi$ from $\pi$. W.l.o.g. the first claim of $\pi$ is $(\emptyset , \emptyset , 1)$ derived with (Ax) and all other claims are not derived with (Ax). We incrementally build a Decision-DNNF and a resolution derivation. For each $k \in [n]$, we have an intermediate Decision-DNNF $D_k$ and resolution derivation $\sigma _k$ that satisfy the following invariants:

(i) $D_k$ is equivalent to $F_k[\alpha _k]$,
(ii) $D_k$ contains only variables from $F_k[\alpha _k]$, and
(iii) every $0$-gate *N* in $D_k$ is labelled with some clause $C \in \sigma _j, j \le k$ such that for any assignment $\beta \in \langle \textsf{vars}(D_k) \rangle$ that reaches *N*, the clause *C* is falsified by $\beta \cup \alpha _k$.

For the *base case* $k= 1$, $I_1 = (\emptyset , \emptyset , 1)$ is derived with (Ax). We set $D_1$ to a circuit that only contains one $1$-gate, and $\sigma _1 = \emptyset$. It is easy to observe that (i) through (iii) are satisfied. For the *induction step*, we distinguish how $I_k$ is derived.

*Join.* $I_k$ is derived with (Join) from claims $I_i$ and $I_j$, so we have $F_k = F_i \cup F_j$ and $\alpha _k = \alpha _i \cup \alpha _j$. Per induction hypothesis, we have already derived Decision-DNNF s $D_i$ and $D_j$ representing $F_i[\alpha _i]$ and $F_j[\alpha _j]$. We define $D_k$ to be an And-gate with the two children that are the roots of $D_i$ and $D_j$. Because of (J-2) we have $\textsf{vars}(F_i) \cap \textsf{vars}(F_j) \subseteq \textsf{vars}(\alpha _i) \cap \textsf{vars}(\alpha _j)$. Together with part (ii) of the induction hypothesis, we get $\textsf{vars}(D_i) \cap \textsf{vars}(D_j) \subseteq \textsf{vars}(F_i[\alpha _i]) \cap \textsf{vars}(F_j[\alpha _j]) = \emptyset$. Therefore, the And-gate is decomposable, so $D_k$ is indeed a Decision-DNNF. We set $\sigma _k = \emptyset$.

To show that (i) is satisfied, we use that $D_i, D_j$ represent $F_i[\alpha _i], F_j[\alpha _j]$ per induction hypothesis. Further, we have $(F_i \cup F_j)[\alpha _i \cup \alpha _j] = F_i[\alpha _i] \cup F_j[\alpha _j]$ by conditions (J-1) and (J-2) for (Join). Therefore, for any assignment $\beta \in \langle \textsf{vars}(F_k[\alpha _k]) \rangle$ holds

$$\begin{aligned} \beta \models F_k[\alpha _k]&\Leftrightarrow \beta \models (F_i \cup F_j)[\alpha _i \cup \alpha _j]\\&\Leftrightarrow \beta \models F_i[\alpha _i] \cup F_j[\alpha _j] \\&\Leftrightarrow (\beta \models F_i[\alpha _i] \text{ and } \beta \models F_j[\alpha _j])\\&\Leftrightarrow (\beta \models D_i \text{ and } \beta \models D_j)\\&\Leftrightarrow \beta \models D_k. \end{aligned}$$

For (ii) we observe:

$$\begin{aligned} \textsf{vars}(D_k)&= \textsf{vars}(D_i) \cup \textsf{vars}(D_j) \\&\subseteq \textsf{vars}(F_i[\alpha _i]) \cup \textsf{vars}(F_j[\alpha _j]) \\&= \textsf{vars}(F_i[\alpha _i] \cup F_j[\alpha _j]) \\&= \textsf{vars}((F_i\cup F_j)[\alpha _i\cup \alpha _j]) \\&= \textsf{vars}(F_k[\alpha _k]). \end{aligned}$$

For (iii), let *N* be some $0$-gate in $D_k$ labelled with *C*, and $\beta$ an assignment that reaches *N*. We assume w.l.o.g. that its path goes through the root of $D_i$. Then $\beta |_{\textsf{vars}(D_i)}$ reaches *N* starting from the root of $D_i$, and according to the induction hypothesis, *C* is falsified by $\beta |_{\textsf{vars}(D_i)} \cup \alpha _i \subseteq \beta \cup \alpha _k$. Therefore, *C* is falsified by $\beta \cup \alpha _k$. Note that $D_k$ contains only one additional gate compared to all previous circuits.

*Composition.* $I_k$ is derived with (Comp) from claims $I_{i_1}, \dots , I_{i_r}$. If $r=0$, i.e., (Comp) uses no previous claims, then we set $D_k$ to be the circuit with only a single $0$-gate. It is easy to see that (i) and (ii) are satisfied. For (iii), let $\rho$ be the absence of models statement that was used to derive $I_k$, it is a derivation $F_k \cup \alpha _k \vdash \square$. If we weaken every clause in this derivation by clause $\overline{\alpha }_k$, we get a resolution derivation $\rho '$ deriving $F_k \vdash \overline{\alpha }_k$. Using the fact that $F_k \subseteq \varphi$, we set $\sigma _k = \rho '$ and use $\overline{\alpha }_k$ as label for the gate.

Otherwise, let $r > 0$, i.e., (Comp) uses at least one claim $(F_{i_1}, \alpha _{i_1})$. Per induction hypothesis, we have the corresponding circuits $D_{i_j}$ for all $j \in [r]$. Let $V = \textsf{vars}(\alpha _{i_1}) \setminus \textsf{vars}(\alpha _k)$, keeping in mind that all assumptions $\alpha _{i_j}$ have the same set of variables because of (C-1). We build a complete binary decision tree *T* with variables in *V*. For every claim $I_{i_j}$ for $j \in [r]$ there is exactly one leaf in *T* that is consistent with the assumption $\alpha _{i_j}$. We replace this leaf with the root of the corresponding Decision-DNNF $D_{i_j}$. Afterwards, we replace all remaining leaves with a $0$-gate. Furthermore, we remove every Decision-gate where both decisions lead to a $0$-gate as long as such gates exist. We set $D_k$ to be the resulting circuit. Note that $D_k$ has at most *r* paths from the root to some $D_{i_j}$ and every such path has at most $|\textsf{vars}(\varphi )|$ Decision-gate s, so the total number of additional Decision-gate s is at most $r \cdot |\textsf{vars}(\varphi )|$. Every Decision-gate can have at most one additional $0$-gate as a child, so the total number of additional $0$-gate s is also bounded by $r \cdot |\textsf{vars}(\varphi )|$.

Per construction, $D_k$ contains exactly the models of $F_k[\alpha _k]$ and $D_k$ contains only variables from $F_k[\alpha _k]$, so (i) and (ii) hold. Finally, we have to find certificates for the 0-gates. There is an absence of models statement $\rho$ deriving $\alpha _k \cup F_k \cup \{\overline{\alpha _{i_j}} \mid j \in [r]\} \vdash \square$. Let *N* be a $0$-gate and $\gamma$ the partial assignment consisting of the decisions on the path from the root of $D_k$ to *N*. Because this path does not lead to any of the $D_{i_j}$, we know that $\gamma$ falsifies all $\alpha _{i_j}$. We obtain $\rho _N$ by weakening every clause in $\rho$ by $\overline{\alpha _k} \vee \overline{\gamma }$. The clauses $(\overline{\alpha _{i_j}} \vee \overline{\alpha _k} \vee \overline{\gamma })$ are tautological and can be removed, so $\rho _N$ derives $F_k \vdash (\overline{\alpha _k} \vee \overline{\gamma })$. We use the clause $(\overline{\alpha _k} \vee \overline{\gamma })$ as the label for *N*, and observe that for every assignment $\beta$ that reaches *N*, $\gamma \subseteq \beta$ and therefore $\beta \cup \alpha _k$ falsifies $(\overline{\alpha _k} \vee \overline{\gamma })$. We set $\sigma _k = \{\rho _N \mid N \text { is a 0-gate}\}$, instantiating $\rho$ at most $r \cdot |\textsf{vars}(\varphi )|$ times.

*Extension.* $I_k$ is derived with (Ext) from $I_i$. Per induction hypothesis, we have already derived a Decision-DNNF$D_i$ equivalent to $F_i[\alpha _i]$. We set $D_k = D_i$ and $\sigma _k = \emptyset$. By using that $\alpha _k$ satisfies $F_k \setminus F_i$ by (E-3) and $\alpha _k|_{\textsf{vars}(F_i)} = \alpha _i$ by (E-2) we get

$$\begin{aligned} F_k[\alpha _k] = F_i[\alpha _k] = F_i[\alpha _k|_{\textsf{vars}(F_i)}] = F_i[\alpha _i] \end{aligned}$$

leading to (i) and (ii). For (iii) we use the fact that we do not change the circuit, together with $\alpha _i \subseteq \alpha _k$ which means that every clause falsified by $\beta \cup \alpha _i$ is also falsified by $\beta \cup \alpha _k$.

This completes the induction. Since $I_n = (\varphi , \emptyset )$, $D_n$ is a Decision-DNNF representing $\varphi$. Further, all 0-gates have valid certificates which are derived with resolution in $\sigma := \bigcup _{i \in [n]} \sigma _i$. Therefore, we have constructed a valid $\mathsf {{kcps}}^\textsf {{Res}}$ proof $\pi ' = (D_n, \sigma )$.

Finally, we show that the size of $\pi '$ is polynomial in the size of $\pi$. We start with a single node in $D_1$. For every (Join) claim we add one gate, and for every (Comp) claim we add at most $2 \cdot n \cdot |\textsf{vars}(\varphi ) |$ gates. In total, we get $|D_n| \le 2 \cdot n^2 \cdot |\textsf{vars}(\varphi )| + 1$. The resolution derivation $\sigma$ is derived from the absence of models statements. Every refutation is instantiated at most $n^2 \cdot |\textsf{vars}(\varphi ) |$ times, so $|\sigma | \le n^2 \cdot |\textsf{vars}(\varphi )| \cdot |\pi |$. $\square$

We remark that there is a related result in the literature [12], which shows that we can efficiently transform any $\textsf{MICE}$ proof of some formula $\varphi$ into a Decision-DNNF *D* representing $\varphi$. The theorem above strengthens this by showing that we can even derive some set $\sigma$ of clauses such that all $0$-gate s of *D* are $\sigma$-certified.

Next, we observe that $\mathsf {{kcps}}^\textsf {{Res}}$ is indeed a generalisation of $\textsf{kcps}$ since we can write any $\textsf{kcps}$ proof *D* as a $\mathsf {{kcps}}^\textsf {{Res}}$ proof $(\sigma , D)$ where $\sigma$ contains all clauses of $\varphi$.

### Observation 4.2

$\mathsf {{kcps}}^\textsf {{Res}}$ p-simulates $\textsf{kcps}$. Further, the simulation is linear-time.

Now, we efficiently transform a given $\mathsf {{kcps}}^\textsf {{Res}}$ proof of a CNF $\varphi$ into a $\mathsf {CPOG^{Decision\text {-}DNNF}}$ proof. The choice of the Decision-DNNF *D* for the $\textsf{CPOG}$ proof is obvious: we simply copy it from the $\mathsf {{kcps}}^\textsf {{Res}}$ proof. Therefore, we only have to construct a short refutation of $\varphi \models D$.

### Theorem 4.3

$\mathsf {CPOG^{Decision\text {-}DNNF}}$ p-simulates $\mathsf {{kcps}}^\textsf {{Res}}$. Further, the simulation is linear-time.

Before we prove this Theorem, we start with the following lemma:

### Lemma 4.4

Let $\pi = (\sigma , D)$ be a $\mathsf {{kcps}}^\textsf {{Res}}$ proof for $\varphi$ and $\mathcal {E}(D)$ the encoding of *D*. Then, for every gate *N* in *D*, we can efficiently derive a clause $C_N = (\vartheta _{N} \vee C)$ from $\mathcal {E}(D) \wedge \varphi$ where *C* is a clause satisfying the invariant (I): *Any assignment leading to N falsifies C.*

### Proof

We show this claim by induction on the gates of *D* starting at the leaves. In the *base case*, let *N* be a leaf of *D*. If *N* is a $0$-gate, let $C \in \sigma$ be the certificate of *N*. Per construction, we have already derived *C* in the proof $\rho$. We apply a weakening step to *C* leading to $C_N = (\vartheta _{N} \vee C)$, and per definition of the certificate, (I) is satisfied. Otherwise, if the leaf *N* is a $1$-gate, there has to be a unit clause $(\vartheta _{N})$ in our encoding $\mathcal {E}(D)$. Thus, we do not have to derive anything in $\rho$ but simply set $C_N = (\vartheta _{N})$, i.e., *C* is the empty clause which obviously fulfils (I).

In the *induction step*, we want to derive $C_N$ for any inner gate *N*. Let $N_1$ and $N_2$ be the children of *N*. Per induction hypothesis, we have already derived the corresponding clauses $C_{N_1} = (\vartheta _{N_1} \vee C_1)$ and $C_{N_2} = (\vartheta _{N_2} \vee C_2)$. To derive $C_N$, we have to distinguish whether *N* is a Decision-gate or an And-gate.

- Let *N* be a Decision-gate deciding variable *x* leading to $N_1$ if $x=0$ and to $N_2$ if $x=1$. Per induction hypothesis, (I) is satisfied for $N_1$ and $N_2$, i.e., we have $\overline{x} \notin C_1$ and $x \notin C_2$. To determine the relevant clauses in $\mathcal {E}(D)$, we recall that a Decision-gate is a shorthand for a small subcircuit in a d-DNNF. Accordingly, we have leaf gates $N_3 = \overline{x}$ and $N_4 = x$, as well as inner gates $N_5 = (N_1 \texttt { and } N_3)$ and $N_6 = (N_2 \texttt { and } N_4)$. The original gate is $N = (N_5 \texttt { or } N_6)$. By construction, $\mathcal {E}(D)$ contains the clauses $(\overline{\vartheta _{N_1}} \vee \overline{\vartheta _{N_3}} \vee \vartheta _{N_5})$, $(\vartheta _{N_3} \vee x)$, $(\overline{\vartheta _{N_5}} \vee \vartheta _{N})$, $(\overline{\vartheta _{N_2}} \vee \overline{\vartheta _{N_4}} \vee \vartheta _{N_6})$, $(\vartheta _{N_4} \vee \overline{x})$ and $(\overline{\vartheta _{N_6}} \vee \vartheta _{N})$. For $C = ((C_1\setminus \{x\}) \vee (C_2\setminus \{\overline{x}\}))$ we derive $C_N = (\vartheta _{N} \vee C)$ starting as follows: 
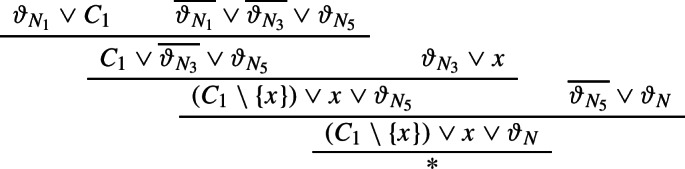
 and finishing as follows: 
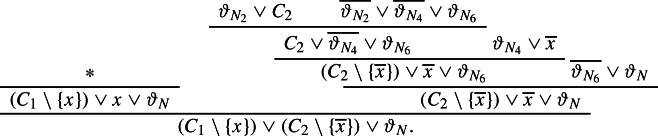
 Finally, we have to argue that *C* satisfies invariant (I). Per induction hypothesis, $C_1$ satisfies (I) for the gate $N_1$, i.e., every path leading to $N_1$ falsifies $C_1$ and in particular $(C_1\setminus \{x\})$. Similarly, every path leading to $N_2$ falsifies $C_2$ and in particular $(C_2\setminus \{\overline{x}\})$. As *N* decides *x*, any path leading to *N* has to falsify $(C_1\setminus \{x\})$ and $(C_2\setminus \{\overline{x}\})$ and therefore *C*.
- Let *N* be an And-gate. By construction, $\mathcal {E}(D)$ contains the clause $(\overline{\vartheta _{N_1}} \vee \overline{\vartheta _{N_2}} \vee \vartheta _{N})$. For $C = (C_1 \vee C_2)$, we derive $C_N = (\vartheta _{N} \vee C)$ as follows: 
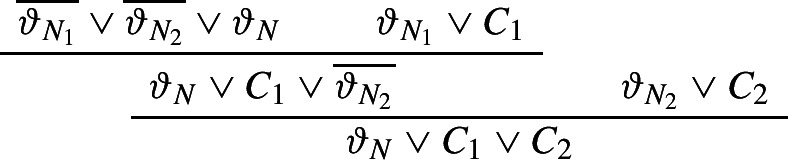
 Finally, we argue that *C* satisfies (I). Per induction hypothesis, $C_1$ satisfies (I) for gate $N_1$, i.e., every path leading to $N_1$ falsifies $C_1$. As *N* does not decide a variable, every path leading to *N* has to falsify $C_1$ as well. Analogously, we see that every path leading to *N* has to falsify $C_2$ and thus also $(C_1 \vee C_2)$.

This completes the induction and finishes the proof of the claim. $\square$

### Proof of Theorem 4.3

Let $\pi = (\sigma , D)$ be a $\mathsf {{kcps}}^\textsf {{Res}}$ proof for $\varphi$. Further, let $\mathcal {E}(D)$ be the clausal Tseitin encoding of the Decision-DNNF *D* with root *R*. For any resolution refutation $\rho$ of $\varphi \wedge \mathcal {E}(D) \wedge (\overline{\vartheta _{R}})$, we obtain a valid $\mathsf {CPOG^{Decision\text {-}DNNF}}$ proof $\pi ' = (\mathcal {E}(D), \rho )$. In order to prove the theorem, we construct $\rho$ such that $|\pi ' | = O(|\pi |)$. As $|\pi ' | = |\mathcal {E}(D) | + |\rho | = O(|D |) + |\rho |$ it is sufficient that $|\rho | = O(|D | + |\sigma |)$. For that, we first derive all clauses of $\sigma$ in $\rho$.

Using Lemma 4.4, we can also derive the clause $C_R = (\vartheta _{R} \vee C)$ for the root *R* such that *C* satisfies invariant (I) from that lemma. As there are no decisions above *R*, *C* has to be the empty clause, i.e., we have derived the unit clause $C_R = (\vartheta _{R})$. By applying a resolution step with the other unit clause $(\overline{\vartheta _{R}})$, we refute $\varphi \wedge \mathcal {E}(D) \wedge (\overline{\vartheta _{R}})$ with resolution.

In total, $\rho$ contains the derivations of $\sigma$ and additionally, a constant number of resolution steps for each gate in *D*. Thus, the resulting resolution refutation $\rho$ has at most size $|\sigma | + O(|D |)$ and the theorem follows. $\square$

We finally show last simulation which almost follows by definition as $\textsf{POG}$ s generalise Decision-DNNF s.

### Observation 4.5

$$\textsf{CPOG}$$ p-simulates $$\mathsf {CPOG^{Decision\text {-}DNNF}}$$.

### Proof

Let $(\mathcal {E}(D), \rho )$ be a $\mathsf {CPOG^{Decision\text {-}DNNF}}$ proof for some formula $\varphi$. *D* is already a $\textsf{POG}$, as any Decision-gate $N = (\texttt {if }x\texttt { then }N_1\texttt { else }N_2)$ is just a simpler notation for $N = (M_1\texttt { or }M_2)$ with $M_1 = (N_1\texttt { and }x)$ and $M_2 = (N_2\texttt { and }\overline{x})$. Therefore, we can copy the Decision-DNNF *D* into a $\textsf{POG}$ as required for Item 1 in Definition 3.6. The proof $\rho$ for Item 2 of Definition 3.6 is analogously. To construct the proof $\psi$ for Item 3 we use Lemma 3.10.

It remains to construct the set *X* as per Item 4 of Definition 3.6 that shows that all the resulting Or-gate s are deterministic. For this, we include the following refutation of $\mathcal {E}(D) \wedge (\vartheta _{M_1}) \wedge (\vartheta _{M_2})$ to *X* for any such Or-gate *N*: 
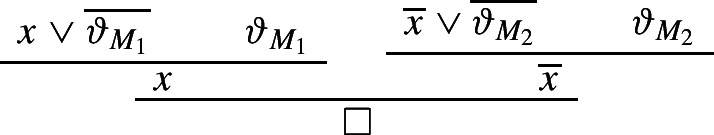


In the end, $(\mathcal {E}(D), \rho , X)$ is a $\textsf{CPOG}$ proof of $\varphi$ with size at most $8 \cdot |(\mathcal {E}(D), \rho ) |$ since *X* contains 7 clauses for every Decision-gate in *D*. $\square$

With that, all simulations illustrated in Fig. 2 are established. Upon closer examination, all these new simulations turn out to be computable with logarithmic space. Moreover, the simulations in Observation 4.2 and Theorem 4.3 can be computed in linear time.

## MICE and kcps are Incomparable

Having determined the simulation order of #SAT proof systems, we now turn to lower bounds and separations between them. We first compare $\textsf{MICE}$ and $\textsf{kcps}$.

### CNFs That are Hard for Kcps But Easy for MICE

Before getting to specific lower bounds, we provide a tight characterisation of proof size on unsatisfiable formulas for $\textsf{kcps}$ in terms of regular resolution.

#### Proposition 5.1

For unsatisfiable formulas, $\textsf{kcps}$ and regular resolution are p-equivalent.

In order to proof this proposition, we start with the following lemma:

#### Lemma 5.2

Let $\varphi$ be an unsatisfiable formula and *D* be a $\varphi$-certified Decision-DNNF representing $\varphi$. Then, there is a $\varphi$-certified Decision-DNNF $D'$ with $D' \equiv \varphi$ and $|D' | \le |D |$ that contains no And-gate s.

#### Proof

To prove this claim we present a technique to remove an And-gate. Let *N* be an And-gate of *D* such that all ancestors of *N* are Decision-gate s. Further, let $N_1$ and $N_2$ be the children of *N*, i.e., $D(N) \equiv D(N_1) \wedge D(N_2)$.

For the sake of contradiction, assume that both $D(N_1)$ and $D(N_2)$ are satisfiable, i.e., there are two satisfying assignments $\alpha _1 \in \langle \textsf{vars}(D(N_1))\rangle$ and $\alpha _2 \in \langle \textsf{vars}(D(N_2))\rangle$. By the decomposability property we have that $\textsf{vars}(D(N_1))$ and $\textsf{vars}(D(N_2))$ are disjoint. Therefore, $\alpha _1 \cup \alpha _2$ is a valid assignment that satisfies $D(N_1) \wedge D(N_2) \equiv D(N)$.

By choice of *N*, the gate *N* has only Decision-gate s as ancestors, hence there is an assignment $\alpha$ that leads to *N*. Because each path through a Decision-DNNF decides each variable at most once, we have $\textsf{vars}(\alpha ) \cap \textsf{vars}(D(N)) = \emptyset$. Therefore, $\alpha \cup \alpha _1 \cup \alpha _2$ is a valid assignment that satisfies $\varphi$ contradicting the unsatisfiability of $\varphi$.

Thus, our assumption that both $D(N_1)$ and $D(N_2)$ are satisfiable leads to a contradiction, so we can assume w.l.o.g. that $D(N_1)$ is unsatisfiable. Therefore $D(N) \equiv D(N_1) \wedge D(N_2) \equiv D(N_1)$. Thus, we can decrease the number of And-gate s of *D* by 1 by replacing gate *N* with $N_1$. This can never increase the set of assignments that reach a particular gate, and therefore leaves all certificates intact. In this way, we can remove every And-gate one by one without increasing the size of *D*. $\square$

#### Proof of Proposition 5.1

The proof is based on [47, Theorem 18.1] stating that the minimal size of any regular resolution refutation of a formula $\varphi$ equals the minimal size of any read-once branching program solving the search problem for $\varphi$. A read-once branching program for the search problem for $\varphi$ is equivalent to a $\varphi$-certified Decision-DNNF *D* with $D \equiv \varphi$ that contains no And-gate s. Thus, the result directly implies that $\textsf{kcps}$ p-simulates regular resolution for unsatisfiable formulas.

For the converse simulation of $\textsf{kcps}$ by regular resolution we consider an arbitrary $\varphi$-certified Decision-DNNF *D* with $D \equiv \varphi$ for some unsatisfiable formula $\varphi$. By using Lemma 5.2, we convert *D* to some $D'$ without And-gate s, apply the result from [47, Theorem 18.1] and obtain a regular resolution refutation of size at most $|D |$. $\square$

Therefore, any lower (and upper) bound for regular resolution transfers to $\textsf{kcps}$. For regular resolution, many lower bounds are known [53], and in particular all formulas hard for resolution such as the pigeonhole principle [41] are hard for $\textsf{kcps}$. Note that any unsatisfiable formula has a trivial Decision-DNNF. Nevertheless, all $\textsf{kcps}$ proofs can be of exponential size. This answers an open question from [21].

A similar proof size characterisation on unsatisfiable formulas is known for $\textsf{MICE}$, in this case in terms of full (i.e. unrestricted) resolution [12].

#### Proposition 5.3

([12]) For unsatisfiable formulas, $\textsf{MICE}$ and resolution are p-equivalent.

As there are CNF families exponentially separating regular and full resolution [1, 69], Propositions 5.1 and 5.3 yield:

#### Corollary 5.4

$\textsf{MICE}$ is exponentially separated from $\textsf{kcps}$.

While this separation is on unsatisfiable formulas, we can easily extend it to satisfiable CNFs as well. For this, let $\varphi$ be an unsatisfiable formula that separates resolution from regular resolution. For some fresh variable $a \notin \textsf{vars}(\varphi )$, we define $\varphi ' = \{ (C \vee a) \mid C \in \varphi \}$. Then, $\varphi '$ has $2^{|\textsf{vars}(\varphi ) |}$ models and still separates $\textsf{MICE}$ from $\textsf{kcps}$.

Firstly, $\varphi '$ is still easy for $\textsf{MICE}$. We derive the claim $(\varphi ', \{\overline{a}\})$ with (Comp), the absence of models statement is short since $\varphi '[\overline{a}] = \varphi$ has a short resolution refutation. Further, we derive $(\varphi ', \{a\})$ with (Ext) and finally apply (Comp) to these two claims, resulting in $(\varphi ', \emptyset )$.

Secondly, we argue that the hardness of $\varphi$ for $\textsf{kcps}$ implies the hardness of $\varphi '$. For the contrapositive, let $D \equiv \varphi '$ be a $\varphi '$-certified Decision-DNNF. Then, $D[\overline{a}] \equiv \varphi$ is a $\varphi$-certified Decision-DNNF of size at most |*D*|, i.e., $\varphi$ has a $\textsf{kcps}$ proof of analogous size.

### CNFs That are Hard for MICE But Easy for Kcps

Next, we show that $\textsf{MICE}$ cannot simulate $\textsf{kcps}$. For that, we use a variant of the pebbling formulas on pyramidal graphs. We show that these formulas have polynomial-sized $\textsf{kcps}$ proofs while any $\textsf{MICE}$ proof is of exponential size.

**Fig. 9:**
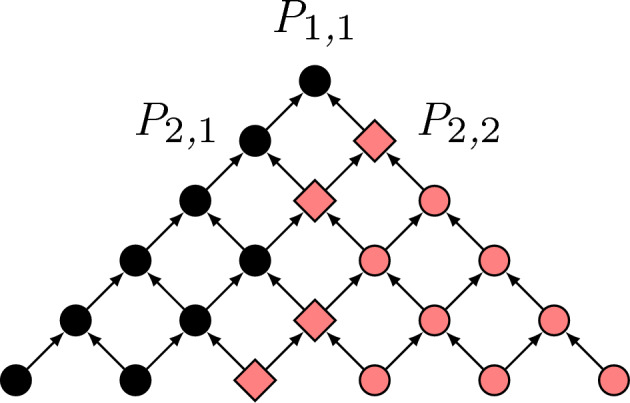
Pyramidal graph for $\textsf {PEB} _6$ consisting of nodes $P_{1,1}, \dots P_{6,6}$, depicting the situation in the proof of Theorem 5.7. For a fixed claim in a $\textsf{MICE}$ proof of $\textsf {PEB} _6$, the red nodes are *active*, i.e., they correspond to variables that occur in the formula *F*. The diamond-shaped nodes form the *boundary* of this claim as they have neighbours that are not active

These pebbling formulas are based on pyramidal graphs (cf. Fig. 9). For a given size $n \in \mathbb {N}$, this graph has $m:= \frac{n(n+1)}{2}$ nodes: a node $P_{i,j}$ for each $1 \le j \le i \le n$. For each $i < n$, there are edges from $P_{i+1,j}$ and $P_{i+1,j+1}$ to $P_{i,j}$. The variable *i* is called the *row* of the node, and *j* is called the *column*. When comparing rows, we talk about *greater* or *smaller* rows. The nodes in row *n* are called sources, and the node in row 1 is called the sink.

We start with some intuition for the pebbling formulas $\textsf {PEB} _n$. They have two variables $w_{i,j}$ and $b_{i,j}$ for each node $P_{i,j}$. $w_{i,j}$ represents a white pebble being placed on that node, while $b_{i,j}$ represents a black pebble. The formula requires each source node to contain a pebble. Every other node needs to contain a pebble if and only if both its parent nodes contain a pebble. No node can simultaneously contain a black and a white pebble.

#### Definition 5.5

Let *n* be an integer. The formula $\textsf {PEB} _n$ has variables $w_{i,j}$ and $b_{i,j}$ for every $i,j \in [n]$ with $j \le i$. $\textsf {PEB} _n$ is a CNF defined as follows:

- For every $i,j \in [n-1], j \le i$ the formula requires that
  $$\begin{aligned} (w_{i,j} \vee b_{i,j}) \leftrightarrow ((w_{i+1,j} \vee b_{i+1,j}) \wedge (w_{i+1,j+1} \vee b_{i+1,j+1})). \end{aligned}$$
  This is expressed using the clauses
  $$\begin{aligned} C_{i,j}^1&= \overline{w_{i+1,j}} \vee \overline{w_{i+1,j+1}} \vee w_{i,j} \vee b_{i,j} \qquad&\qquad C_{i,j}^2&= \overline{w_{i+1,j}} \vee \overline{b_{i+1,j+1}} \vee w_{i,j} \vee b_{i,j} \\ C_{i,j}^3&= \overline{b_{i+1,j}} \vee \overline{w_{i+1,j+1}} \vee w_{i,j} \vee b_{i,j} \qquad&\qquad C_{i,j}^4&= \overline{b_{i+1,j}} \vee \overline{b_{i+1,j+1}} \vee w_{i,j} \vee b_{i,j} \\ C_{i,j}^5&= w_{i+1,j} \vee b_{i+1,j} \vee \overline{w_{i,j}} \qquad&\qquad C_{i,j}^6&= w_{i+1,j} \vee b_{i+1,j} \vee \overline{b_{i,j}} \\ C_{i,j}^7&= w_{i+1,j+1} \vee b_{i+1,j+1} \vee \overline{w_{i,j}} \qquad&\qquad C_{i,j}^8&= w_{i+1,j+1} \vee b_{i+1,j+1} \vee \overline{b_{i,j}}. \end{aligned}$$
- For every $j \in [n]$ there is a clause $w_{nj} \vee b_{nj}$.
- For every $i,j \in [n], j \le i$ there is a clause $C_{i,j}^9 = \overline{b_{i,j}} \vee \overline{w_{i,j}}$.

Note that the commonly used pebbling formulas require the sink node $P_{1,1}$ to contain no pebbles, making the formula unsatisfiable. We omit this requirement and obtain a formula that is satisfied if and only if each node contains exactly one pebble. It has $2^m$ models where *m* is the number of nodes. Two nodes are called *adjacent* if there is an edge between them in the pebbling graph.

To separate $\textsf{kcps}$ from $\textsf{MICE}$ with $\textsf {PEB} _n$, we show that there are polynomial-sized proofs in $\textsf{kcps}$ while any $\textsf{MICE}$ proof requires exponential size. We start with the upper bound.

#### Proposition 5.6

There are $\textsf{kcps}$ proofs for the $\textsf {PEB} _n$ formulas of size $O(|\textsf {PEB} _n |)$.

#### Proof

We iteratively construct a $\textsf {PEB} _n$-certified Decision-DNNF *D* with $D \equiv \textsf {PEB} _n$. For each node $P_{i,j}$, we construct a partial Decision-DNNF with root $N_{i,j}$ that handles the case $\{w_{i,j} = 0, b_{i,j} = 0\}$. This means that in order to obtain a valid Decision-DNNF, all paths to $N_{i,j}$ must include these two decisions. We also make sure that descendants of $N_{i,j}$ only decide variables of nodes in rows greater than *i*.

We begin constructing the $N_{i,j}$ for greater rows, starting with $i=n$, and continue to smaller rows. For $i = n$, $N_{i,j}$ is simply a $0$-gate labelled with the clause $w_{n,j} \vee b_{n,j}$, which will be falsified by the assumption $\{w_{i,j} = 0, b_{i,j} = 0\}$.

**Fig. 10:**
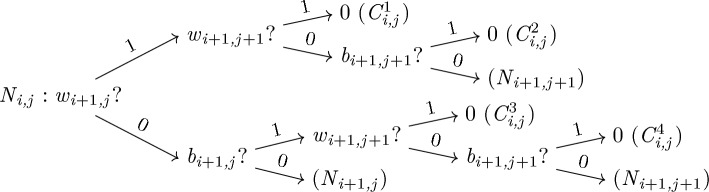
Fragment for the Decision-DNNF in Proposition 5.6. The $0$-gate s are certified with clauses $C_{i,j}$ from Definition 5.5

For $i < n$, we add $N_{i,j}$ according to Fig. 10. The leaves of this proof fragment are either $0$-gate s that are certified by some clause $C_{i,j}^1$ to $C_{i,j}^4$, or are connected to some previously constructed gate $N_{i+1,j}$ or $N_{i+1,j+1}$, after making sure that the corresponding node contains no pebbles. In this way, we can obtain a graph that contains an appropriate gate $N_{i,j}$ for every node $P_{i,j}$.

Finally, we build the complete Decision-DNNF *D*. For each node $P_{i,j}$, ordered from least to greatest *i*, we decide $w_{i,j}$ and, if it is 0, also $b_{i,j}$. If both are 0, we connect it to $N_{i,j}$; if both are 1, we connect it to a $0$-gate certified by $C_{i,j}^9$. We merge the branches of all other cases and continue with the next node. After all nodes have been handled, we finally arrive at a single $1$-gate.

Because of the ordering of the nodes, each path through *D* can decide each variable at most once. Therefore, *D* is indeed a Decision-DNNF. It is equivalent to $\varphi$ and $\varphi$-certified. For each node $P_{i,j}$ we add at most 13 gates, and there is one additional $1$-gate. In total, the number of gates is at most $13m+1 = O(|\textsf {PEB} _n |)$. $\square$

The actual lower bound for $\textsf{MICE}$ is the more challenging part.

#### Theorem 5.7

The $\textsf {PEB} _n$ formulas require $\textsf{MICE}$ proofs of size $2^{\Omega (n)}$.

Note that all known lower bounds for $\textsf{MICE}$ so far are based on formulas with large Decision-DNNF representation [12]. However, this lower bound technique does not work for $\textsf {PEB} _n$ as it has polynomial-sized Decision-DNNF s (Proposition 5.6).

#### Proof

In this proof we state some claims that we prove separately afterwards for better readability. Let $\pi$ be a $\textsf{MICE}$ proof of $\textsf {PEB} _n$. We start with some notions. For a claim $I = (F, \alpha )$, we define set of *active* nodes $\mathcal {A}(I)$ as

$$\begin{aligned} \mathcal {A}(I) := \{P_{i,j} \mid w_{i,j} \in \textsf{vars}(F) \text { or } b_{i,j} \in \textsf{vars}(F)\}, \end{aligned}$$

the *width* *w*(*I*) as

$$\begin{aligned} w(I) = |\mathcal {A}(I) |, \end{aligned}$$

and the *boundary* *B*(*I*) as

$$\begin{aligned} B(I) := \{N \in \mathcal {A}(I) \mid \text {there is an adjacent node } N' \notin \mathcal {A}(I)\}. \end{aligned}$$

Any claim that considers about half of all nodes also needs to have a large boundary:

#### Claim 5.8

Let *I* be a claim which width is bounded by $\frac{m}{3} \le w(I) \le \frac{2 \cdot m}{3}$. Then, $|B(I) | \ge \frac{n}{8}$.

Let $G_\pi = (V,E)$ be the representation of $\pi$ as proof graph, i.e., *V* is the set of all claims in $\pi$ and there is an edge $(I_1, I_2)$ between two claim exactly if $I_1$ was used to derive $I_2$. Any claim $I \in V$ that is derived with (Join) has two incoming edges. For any such node, we delete the edge from the child that has the smaller width. We refer to the resulting graph as $G'_\pi = (V, E')$.

#### Claim 5.9

For every model $\beta \models \textsf {PEB} _n$, there is a path $\pi _{\beta }$ from the final claim to an (Ax) claim, along edges in $E'$, such that for every every claim $(F, \alpha ) \in \pi _\beta$ holds $\beta \models \alpha$.

In the rest of the proof, we use the $\pi _\beta$ from Claim 5.9 and define $V'$ to be the union of all $\pi _\beta$. Claims in $V'$ with a large boundary also have a large assumption:

#### Claim 5.10

Any claim $I = (F, \alpha ) \in V'$ satisfies $|\alpha | \ge |B(I) |$.

Next, we partition the claims of $V'$ into two sets

$$\begin{aligned} X&= \{I \in V' \mid w(I) < \frac{2}{3} \cdot m\}, \\ Y&= \{I \in V' \mid w(I) \ge \frac{2}{3} \cdot m\}. \end{aligned}$$

Further, we define the set $S \subseteq Y$ as the set of nodes in *Y* that have a child in *X*, i.e.

$$\begin{aligned} S = \{I \in Y \mid \exists I_1 \in X: (I_1, I) \in E'\}. \end{aligned}$$

All claims in *S* have large assumptions:

#### Claim 5.11

Any claim $I = (F,\alpha ) \in S$ satisfies $|\alpha | \ge \frac{n}{8}$.

On the other hand, every model of $\textsf {PEB} _n$ corresponds to a claim in *S*:

#### Claim 5.12

Let $\beta$ be a model of $\textsf {PEB} _n$. Then, there is a claim $(F,\alpha ) \in S$ such that $\alpha$ and $\beta$ are consistent.

Using Claims 5.11 and 5.12, we can finally prove the lower bound for the theorem. It is easy to observe that $\textsf {PEB} _n$ has $2^m$ models because there are 2 satisfying assignments to the variables of each node. Let $\beta$ be an assumption with at least $\frac{n}{8}$ variables and *s* the number of nodes with one or two variables in $\beta$. We observe that $2s \ge \frac{n}{8}$ and the number of models consistent with $\beta$ is at most $1^{s} \cdot 2^{m-s} \le 2^m \cdot 2^{n / 16}$. Because each of $2^m$ models is consistent with a claim in *S*, *S* has at least $2^{n / 16}$ elements. We conclude that $|\pi | \ge |V' | \ge |S | \ge 2^{n / 16} = 2^{\Omega (n)}$ leading to the theorem. $\square$

Finally, we prove the five used claims.

#### Claim 5.8

Let *I* be a claim which width is bounded by $\frac{m}{3} \le w(I) \le \frac{2 \cdot m}{3}$. Then, $|B(I) | \ge \frac{n}{8}$.

#### Proof

**Fig. 11:**
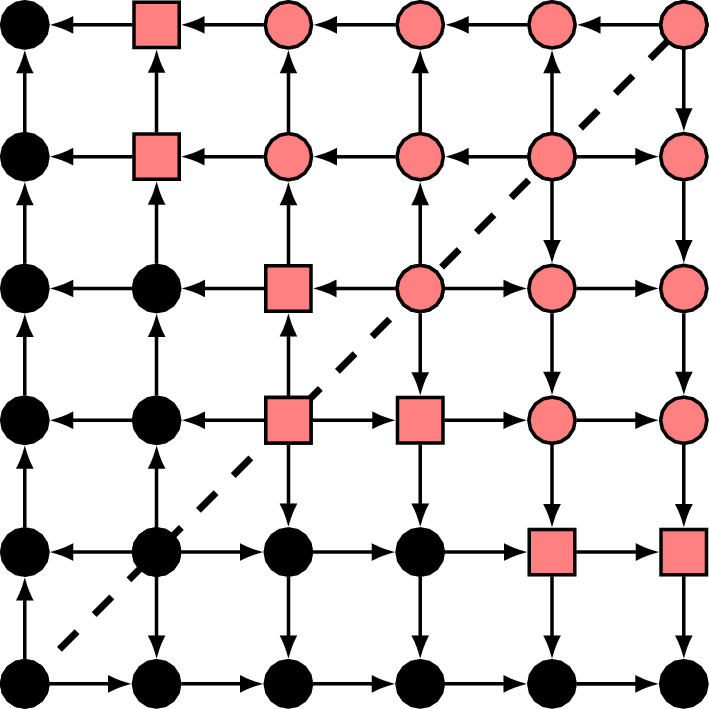
The proof of Claim 5.8 is based on a duplicated pyramidal graph from Fig. 9

For $n < 8$ the statement is trivial, so we can assume $n \ge 8$. We mirror the nodes in Fig. 9 along the bottom row to obtain a square grid *G* with $n^2$ nodes and edges of length *n* which is illustrated in Fig. 11. Each of the *n* source nodes is its own mirror image, the other nodes are duplicated. A mirror node is considered active if it is the mirror image of an active node. Note that the number of boundary nodes in *G* is at most $2 \cdot |B(I) |$. We prove the statement by showing that *G* contains at least $\frac{n}{4}$ boundary nodes.

The original graph contains at least $\frac{1}{3} \cdot m = \frac{n(n+1)}{6}$ active nodes. At most *n* of them are source nodes in the bottom row, the rest will be duplicated in *G*. Therefore, *G* contains at least $2 \cdot \frac{n(n+1)}{6} - n$ active nodes. For $n \ge 8$, this is at least $\frac{1}{4}n^2$. The same argument holds for the number of inactive nodes in *G*.

We view *G* in terms of rows and columns of length *n*, effectively rotating it by $45^\circ$ compared to Fig. 9. A row that contains both active and inactive nodes must contain at least one boundary node. If there are $\frac{n}{4}$ or more of these rows, we are finished. Otherwise, the mixed rows contain fewer than $\frac{1}{4}n^2$ nodes. Because there are at least $\frac{1}{4}n^2$ inactive nodes, at least one inactive node has to be in a row without any active nodes, so there is a row with only inactive nodes. For analogous reasons, there is a row with only active nodes. Consequently, every column contains both active and inactive nodes, and must therefore contain at least one boundary node, for a total of at least *n* boundary nodes. $\square$

#### Claim 5.9

For every model $\beta \models \textsf {PEB} _n$, there is a path $\pi _{\beta }$ from the final claim to an (Ax) claim, along edges in $E'$, such that for every every claim $(F, \alpha ) \in \pi _\beta$ holds $\beta \models \alpha$.

#### Proof

We fix some $\beta$ and show that any claim $I \in V$ that has an assumption consistent with $\beta$ has a child $I_1 \in \pi$ (according to $E'$) whose assumption is consistent with $\beta$ as well. We can then build the path by starting at the final claim and repeatedly moving to that child until we arrive at an (Ax) claim. We distinguish how *I* is derived.

- If *I* is derived with (Ax), we are finished.
- *I* is derived with (Comp) using claims $I_1, \dots , I_k$. Since there is a path from *I* to the root node, $F \subseteq \textsf {PEB} _n$. As $\beta$ is a model of $\textsf {PEB} _n$, it is a model of *F*, too. Therefore, $\beta$ has to be contained in one of the assumptions as there would be no absence of model statement for the (Comp) otherwise.
- *I* is derived with (Join) using claim $I_1$. Per definition of (Join) is $\alpha _1 \subseteq \alpha$, i.e., $\beta$ is also a model of $\alpha _1$.
- *I* is derived with (Ext) using claim $I_1$. Per definition of (Ext) is $\alpha _1 \subseteq \alpha$, i.e., $\beta$ is also a model of $\alpha _1$. $\square$

#### Claim 5.10

Any claim $I = (F, \alpha ) \in V'$ satisfies $|\alpha | \ge |B(I) |$.

#### Proof

Let $I = (F, \alpha ) \in V'$, i.e., there is an assignment $\beta$ with $I \in \pi _\beta$. Let $P_{i,j} \in B(I)$ be a node in the boundary. Because $P_{i,j} \in B(I) \subseteq \mathcal {A}(F)$, at least one of the variables $w_{i,j}, b_{i,j}$ is in $\textsf{vars}(F)$. Assume w.l.o.g. $w_{i,j} \in \textsf{vars}(F)$. We will show that at least one of $w_{i,j}, b_{i,j}$ is in $\textsf{vars}(\alpha )$, by doing this for every node the statement follows. For the sake of contradiction, assume $w_{i,j}, b_{i,j} \notin \textsf{vars}(\alpha )$.

If any clause *C* is in $\textsf {PEB} _n \setminus F$, it has to be added to the formula somewhere along $\pi _\beta$ between *I* and the final claim. If there is a variable $v \in \textsf{vars}(C) \cap \textsf{vars}(F) \setminus \textsf{vars}(\alpha )$, there is no rule that allows it to leave the formula or join the assumption in a subsequent claim. This means that $v \in \textsf{vars}(C) \cap \textsf{vars}(F') \setminus \textsf{vars}(\alpha ')$ for every subsequent claim $I' = (F', \alpha ')$, and therefore *C* cannot be added to the formula in a (Join) step. The only rule that can add *C* to the formula is therefore (Ext). To achieve that, the assumption in that (Ext) claim must satisfy *C*, and specifically a literal $l \in C$. Therefore, $l \in \beta$. Also, $\textsf{var}(l)$ cannot be in $\textsf{vars}(F) \setminus \textsf{vars}(\alpha )$, since that would prevent it from ever being added to an assumption in a later claim. To summarise, any clause $C \in \textsf {PEB} _n \setminus F$ with $\textsf{vars}(C) \cap \textsf{vars}(F) \setminus \textsf{vars}(\alpha ) \ne \emptyset$ must contain a literal *l* with $l \in \beta$ and $\textsf{var}(l) \notin \textsf{vars}(F) \setminus \textsf{vars}(\alpha )$.

$P_{i,j}$ has a neighbouring node not in $\mathcal {A}(F)$, and we distinguish whether this is its parent or child.

- If the inactive node neighbouring $P_{i,j}$ is its parent, we assume w.l.o.g. that it is its left parent, i.e., $P_{i+1,j} \notin \mathcal {A}(F)$ and thus $w_{i+1,j}, b_{i+1,j} \notin \textsf{vars}(F)$. We recall that
  $$\begin{aligned} C_{i,j}^1&= \overline{w_{i+1,j}} \vee \overline{w_{i+1,j+1}} \vee w_{i,j} \vee b_{i,j} \quad&\quad C_{i,j}^2&= \overline{w_{i+1,j}} \vee \overline{b_{i+1,j+1}} \vee w_{i,j} \vee b_{i,j} \\ C_{i,j}^3&= \overline{b_{i+1,j}} \vee \overline{w_{i+1,j+1}} \vee w_{i,j} \vee b_{i,j} \quad&\quad C_{i,j}^4&= \overline{b_{i+1,j}} \vee \overline{b_{i+1,j+1}} \vee w_{i,j} \vee b_{i,j}. \end{aligned}$$
  This means that $C_{i+1,j}^t \notin F$ for $t \in \{1,2,3,4\}$. All these clauses contain $w_{i,j} \in \textsf{vars}(F) \setminus \textsf{vars}(\alpha )$, and must therefore each contain a literal *l* with $l \in \beta$ and $\textsf{var}(l) \notin \textsf{vars}(F) \setminus \textsf{vars}(\alpha )$. Since $\beta$ is a model of the formula, the nodes $P_{i+1,j}$ and $P_{i+1,j+1}$ contain at least one pebble each. Assume w.l.o.g. that both are white, i.e., $\beta (w_{i+1,j}) = \beta (w_{i+1,j+1}) = 1$. This means that the only possible literal *l* for $C_{i,j}^1$ is $b_{i,j}$, so $\beta (b_{i,j}) = 1$ and $b_{i,j} \notin \textsf{vars}(F) \setminus \textsf{vars}(\alpha )$. Since $b_{i,j} \notin \textsf{vars}(\alpha )$, we have $b_{i,j} \notin \textsf{vars}(F)$ and therefore $C_{i,j}^9 = (\overline{b_{i,j}} \vee \overline{w_{i,j}}) \notin F$.
- If the inactive node neighbouring $P_{i,j}$ is its child, we assume w.l.o.g. that it is its right child, i.e., $P_{i-1,j} \notin \mathcal {A}(F)$ and thus $w_{i-1,j}, b_{i-1,j} \notin \textsf{vars}(F)$. We recall that
  $$\begin{aligned} C_{i-1,j}^5&= w_{i,j} \vee b_{i,j} \vee \overline{w_{i-1,j}} \qquad&\qquad C_{i-1,j}^6&= w_{i,j} \vee b_{i,j} \vee \overline{b_{i-1,j}}. \end{aligned}$$
  This means that $C_{i-1,j}^5, C_{i-1,j}^6 \notin F$. Both clauses contain $w_{i,j} \in \textsf{vars}(F) \setminus \textsf{vars}(\alpha )$, and must therefore each contain a literal *l* with $l \in \beta$ and $\textsf{var}(l) \notin \textsf{vars}(F) \setminus \textsf{vars}(\alpha )$. Since $\beta$ is a model of the formula, the node $P_{i-1,j}$ contains at least one pebble. Assume w.l.o.g. it is white, i.e., $\beta (w_{i-1,j}) = 1$. This means that the only possible literal *l* for $C_{i-1,j}^5$ is $b_{i,j}$, so $\beta (b_{i,j}) = 1$ and $b_{i,j} \notin \textsf{vars}(F)$ and therefore $C_{i,j}^9 \notin F$.

In both cases, we saw that $C_{i,j}^9 = (\overline{b_{i,j}} \vee \overline{w_{i,j}}) \notin F$ and $\beta (b_{i,j}) = 1$. Because $w_{i,j} \in \textsf{vars}(C_{i,j}^9) \cap \textsf{vars}(F) \setminus \textsf{vars}(\alpha )$, there has to be a literal *l* in $C_{i,j}$ that is consistent with $\beta$ and $\textsf{var}(l) \notin \textsf{vars}(F) \setminus \textsf{vars}(\alpha )$. There is none, which is a contradiction. $\square$

#### Claim 5.11

Any claim $I = (F,\alpha ) \in S$ satisfies $|\alpha | \ge \frac{n}{8}$.

#### Proof

We distinguish how *I* is derived.

- *I* cannot be derived with (Ax), as $I \in Y$ implies $w(I) \ge n$ and in particular $F \ne \emptyset$.
- *I* cannot be derived with (Comp). For the sake of contradiction, we assume otherwise. Since $I \in S$ there has to be some claim $I_1 \in X$ that was used to derive *I*. Per definition of (Comp), $F = F_1$ which means that *I* has to be in *X* as well which is a contradiction.
- *I* is derived with (Join) using some claims $I_1, I_2$ and w.l.o.g. is $w(I_1) \ge w(I_2)$. Per construction of $G'_\pi$ there the only incoming edge to *I* is from $I_1$. It is easy to observe that $w(I) \le w(I_1) + w(I_2) \le 2 \cdot w(I_1)$. Together with $I \in Y$, i.e., $w(I) \ge \frac{2}{3} \cdot m$, we get $w(I_1) \ge \frac{m}{3}$. However, because $I \in S$ we have $I_1 \in X$ and $w(I_1) < \frac{2}{3} \cdot m$. We apply Claim 5.8 to obtain $|B(I_1) | \ge \frac{n}{8}$. Due to Claim 5.10, the assumption of $I_1$ contains at least $\frac{n}{8}$ variables. When going from $I_1$ to *I*, it cannot decrease. This leads to $|\alpha | \ge \frac{n}{8}$.
- *I* is derived with (Ext) using some claim $I_1$. If $w(I_1) \ge \frac{m}{3}$, we obtain $|\alpha | \ge \frac{n}{8}$ with the exact same argumentation from the previous case. Otherwise, if $w(I_1) < \frac{m}{3}$, we have to introduce variables for at least $|\textsf{vars}(F) | - |\textsf{vars}(F_1) | \ge \frac{2}{3} \cdot m - \frac{1}{3} \cdot m = \frac{m}{3}$ nodes with (Ext), those variables are in $\alpha$. $\square$

#### Claim 5.12

Let $\beta$ be a model of $\textsf {PEB} _n$. Then, there is a claim $(F,\alpha ) \in S$ such that $\alpha$ and $\beta$ are consistent.

#### Proof

We start at the final claim $(\textsf {PEB} _n, \emptyset ) \in Y$ and move along $\pi _\beta$ until we arrive at a claim in *X*. The claim immediately before that one is in *S*. $\square$

By combining Proposition 5.6 and Theorem 5.7, we finally obtain the separation of $\textsf{kcps}$ from $\textsf{MICE}$:

#### Corollary 5.13

$\textsf{kcps}$ is exponentially separated from $\textsf{MICE}$.

While $\textsf{MICE}$ and $\textsf{kcps}$ are incomparable (Corollary 5.4, Corollary 5.13), $\mathsf {{kcps}}^\textsf {{Res}}$ simulates both systems (Theorem 4.1, Observation 4.2), which immediately leads to the following two separations.

#### Corollary 5.14

$\mathsf {{kcps}}^\textsf {{Res}}$ is exponentially separated from $\textsf{MICE}$ and from $\textsf{kcps}$.

With that, we have proven all separations from Fig. 2.

## Conclusion and Future Work

In this paper, we compare the strength of existing proof systems for #SAT. We mention that four of the systems we study, namely $\textsf{MICE}$, $\mathsf {{kcps}}^\textsf {{Res}}$, $\mathsf {CPOG^{Decision\text {-}DNNF}}$ and $\textsf{CPOG}$, include propositional resolution derivations in proofs. These resolution derivations are needed to check propositional entailment steps. We could define variants of the four mentioned proof systems by replacing all resolution proofs by proofs in a different propositional proof system *P* (and in the extreme case even with NP oracle calls). Close inspection of our results shows that all simulations and separations as depicted in Fig. 2 will continue to hold when resolution is replaced throughout by an arbitrary proof system *P* that is at least as strong as resolution (or an NP oracle).

We discuss a few directions for further work. From a *practical perspective*, our simulation results imply that $\textsf{CPOG}$ might indeed be a suitable choice for proof logging as it simulates all other #SAT proof systems. But also $\mathsf {CPOG^{Decision\text {-}DNNF}}$ or $\mathsf {{kcps}}^\textsf {{Res}}$ could be practically sufficient for proof logging for all state-of-the-art #SAT solvers (and as of now, neither of these is known to be strictly weaker than $\textsf{CPOG}$).

In a related direction, we ask whether state-of-the-art knowledge compilers could effectively take advantage of $\mathsf {{kcps}}^\textsf {{Res}}$ by using resolution instead of strictly relying on existing input clauses for certificates. We see this especially in the light that component caching-based #SAT solvers can be directly turned into practically effective knowledge compilers [48]. Hence, one might ask whether we can design even stronger knowledge compilers. Alternatively, we may use $\textsf{kcps}$ or $\textsf{CPOG}$ to certify caching-based #SAT solvers by emitting a Decision-DNNF .

From a *theoretical perspective* the system $\mathsf {{kcps}}^\textsf {{Res}}$ appears quite interesting as it has an easy definition and is still strong enough to capture the different approaches of $\textsf{MICE}$ and $\textsf{kcps}$. Designing a designated lower bound technique for $\mathsf {{kcps}}^\textsf {{Res}}$ appears to be an interesting problem.

## Data Availability

No datasets were generated or analysed during the current study.

## Footnotes

1 However, it is already known that from a $\textsf{MICE}$ proof a Decision-DNNF for the CNF can be extracted [12]. This does not, however, entail a simulation of $\textsf{MICE}$ by $\textsf{kcps}$ (which are based on Decision-DNNF s), and in fact such a simulation *fails* as implied by our separation results in B.
